# Ferroptosis-Related Gene Signature Promotes Ovarian Cancer by Influencing Immune Infiltration and Invasion

**DOI:** 10.1155/2021/9915312

**Published:** 2021-05-26

**Authors:** Yang You, Qi Fan, Jianyun Huang, Yaoqiu Wu, Haiyan Lin, Qingxue Zhang

**Affiliations:** Reproductive Medicine Center, The Sun Yat-sen Memorial Hospital, Sun Yat-sen University, Guangzhou 510120, Guangdong, China

## Abstract

Ovarian cancer is a kind of gynecological malignancy with high mortality. Ferroptosis is a new type of iron-dependent cell death characterized by the formation of lipid peroxides and excessive accumulation of reactive oxygen species. Studies have shown that ferroptosis modulates tumor genesis, progression, and invasion, including ovarian cancer. Based on the mRNA expression data from TCGA, we construct a scoring system using consensus clustering analysis, univariate Cox regression analysis, and least absolute selection operator. Then, we systematically evaluate the relationship between score and clinical characteristics of ovarian cancer. The result from the prediction of biofunction pathways shows that score serves as an independent prognostic marker for ovarian cancer and affects tumor progression by modulating tumor metastasis. Moreover, immunocytes such as activated CD4 T cell, activated CD8 T cell, regulatory T cells, macrophage, and stromal cells, including adipocytes, epithelial cells, and fibroblast infiltrate more in the tumor microenvironment in a high-score group, indicating ferroptosis can also affect tumor immune landscape. Critically, four potentially sensitive drugs, including staurosporine, epothilone B, DMOG, and HG6-64-1 based on the scores, are predicted, and DMOG is recognized as a novel targeted drug for ovarian cancer. In general, we construct the scoring system based on ferroptosis-related genes that can predict the prognosis of ovarian cancer patients and propose that ferroptosis may affect ovarian cancer progression by mediating tumor metastasis and immune landscape. Novel drugs to target ovarian cancer are also predicted.

## 1. Introduction

Ovarian cancer is the most lethal malignancy among gynecological tumors, killing about 150,000 women each year [1]. According to the statistical results of cancer incidence and mortality in the United States in 2020, ovarian cancer mortality ranked fifth in female cancer mortality and ranked first in gynecologic tumor mortality in North America [2]. Due to the lack of typical clinical symptoms in the early stage, 75% of ovarian cancer patients have reached the advanced stage when diagnosed, and 70%–80% of patients relapse after treatment [3]. The 5-year survival rate is about 25%–35%, and the survival rate in most countries has not changed much in the past 20 years [4]. Most of the patients with advanced ovarian cancer develop into relapse and multi-drug resistant stages finally [5]. It has been previously reported that ovarian cancer is divided into four subtypes including differentiated, immunoreactive, proliferative, and mesenchymal subtypes, based on gene expression [6]. Mesenchymal has the highest degree of malignancy, and its invasion ability is the strongest among these four subtypes [7]. In the mesenchymal subtype, the sample has more infiltrating stromal cells [7].

In 2012, Dixon et al. defined an iron-dependent, non-apoptotic cell death induced by erastin as ferroptosis for the first time [8]. Ferroptosis is a novel cell death with characteristics including the production of lipid peroxides and excessive accumulation of lethal reactive oxygen species (ROS) [9]. The key factors of regulating ferroptosis are cysteine-glutamate reverse transporter system (System XC-) and glutathione-dependant peroxidase 4 (GPX4) [10]. System XC- transfers intracellular glutamate to the extracellular matrix and extracellular cysteine to the cytoplasm, where cysteine synthesizes glutathione in the sulfur transduction pathway [11]. Inhibition of System XC- induces ferroptosis [12]. Glutathione is an essential cofactor for GPX4 activation, and its production and maintenance are key to protecting cells from oxidative stress. GPX4 is an enzyme that decomposes H_2_O_2_ and organic H_2_O_2_ into water or corresponding alcohols [13]. Decreased intracellular GPX4 activity or direct degradation of GPX4 can lead to increased iron-dependent reactive oxygen species, then induce ferroptosis [14, 15]. Ferroptosis is also different from apoptosis, autophagy, and necrosis in morphology, metabolism, and biochemistry. Apoptosis is characterized by cell shrinkage, nucleolysis, chromatin agglutination and marginalization, membrane blistering, and the formation of the apoptotic body [16, 17]. The morphological characteristics of autophagy include the formation of autophagosomes in the cytoplasm, a kind of double-membrane vesicle [18, 19]. The cytoplasm and organelles of necrotic cells are swollen, and the cell membrane is ruptured [20]. In terms of the morphological characteristics of ferroptosis, the mitochondrial volume is decreased, but the density of mitochondrial membrane density is increased; the mitochondrial cristae decrease or disappear, but the nuclear size is normal; the chromatin is not condensed; and the cell membrane is intact, so it has the appearance of a complete spherical cell. The occurrence of ferroptosis is closely related to cysteine metabolism [15], lipid metabolism [9], and iron metabolism [21].

Several research works have reported that the activation of ferroptosis is closely associated with tumor progression. Basuli et al. [22] reported significantly more abnormal iron accumulation in high-grade serous ovarian cancer tissues than normal ovarian tissues. Moreover, iron transportation-related genes including TFR1, FPN, and TF were also dysregulated in the ovarian cancer protocell genetic model. Together, ferroptosis may be related to ovarian cancer progression considering the relationship between iron accumulation and ferroptosis. The mutation of p53 and the activation of K-ras have been confirmed as a key regulator of ovarian cancer, including tumorigenesis and tumor migration [23]. P53 targets SLC7A11, the subunit of the System XC-, and inhibits the uptake of cystine to inhibit ferroptosis [24]. According to recent literature reports, PARP inhibition decreases glutathione biosynthesis by downregulating the expression of SLC7A11, thus promotes lipid peroxidation and ferroptosis, and plays a therapeutic role on BRCA-mutant cancer [25]. The above researches suggest that the induction of ferroptosis in ovarian cancer has a therapeutic effect. A multicenter randomized controlled trial that uses sorafenib in combination with topotecan for maintenance therapy reported that a significant improvement in progression-free survival of patients with platinum-resistant ovarian cancer [26] by inducing ferroptosis [27]. These studies indicate that ferroptosis plays an important role in the occurrence, development, and migration of ovarian cancer and that targeting ferroptosis may be a novel strategy for ovarian cancer treatment.

In this study, we aim to investigate the prognostic value of the expression profile of ferroptosis-associated genes (FRGs) in ovarian cancer. We construct a score risk signature model based on FRGs from the TCGA data set, including one RNA sequencing data set and two RNA array data sets, to evaluate the association between ferroptosis and ovarian cancer progression. The workflow was affiliated as Supplementary Figure 1. The score risk signature includes 13 genes such as TGM2, ASAH1, STK3, RIN2, HERC3, FTH1, FMO2, EHD2, COL8A2, C5AR1, AXL, ADORA3, and ADAM9. We find that lymph nodes invasion and venous invasion are more common in the high-score group, and the score is positively correlated with invasion. Moreover, the score is related to immune infiltration. Our results show that the infiltration of immune cells and stromal cells is more in the high-score group. The results of GO and KEGG enrichment analyses indicate that ferroptosis-related biological pathways like cell–cell adhesion mediated by integrin and regulation of extracellular matrix disassembly are activated in the high-score group. The immune cell analysis shows that the expression level of activated CD4^+^ T cell, activated CD8^+^ T cell, regulatory T cells, and macrophage is higher in the high-score group. Critically, we predict staurosporine, epothilone B, DMOG, and HG6-64-1 as potential targeted drugs for ovarian cancer patients.

## 2. Materials and Methods

### 2.1. Data Processing

The expression data of mRNA of ovarian cancer were downloaded from the TCGA websites (https://xenabrowser.net/). All data were transformed into TPM before analyzing. The RNA-microarray AgilentG4502A_07_3 data was set as a training cohort while the RNA-seq data and the RNA-microarray AffyU133a data were applied as a validation cohort.

### 2.2. The Clustering Model

The ferroptosis-related gene was collected from the previous study [28]. Consensus clustering analysis was analyzed with the *R* package “Consensus Cluster Plus,” and samples were classified into cluster 1 or 2. The survival analysis and the ROC curve were performed to view the prognosis ability difference.

### 2.3. Construction of the Scoring System

Differential expressional genes (909 DEGs in total; the screen criteria was 0.677, which was automatically calculated by considering the mean and standard deviation of logFC) between clusters 1 and 2 were identified with *P* packages “limma” [29, 30].

(1) $$\begin{array}{c} \log\text{ FC}=\text{mean}\left(\left|\log\text{ FC}\right|\right)+2*\text{sd}\left(|\log\text{ FC}\right). \end{array}$$

Univariate Cox regression analysis and the LASSO algorithm were applied to discover ovarian cancer prognosis associated genes, and 13 genes were finally filtered out to construct the scoring system.

(2) $$\begin{array}{c} \text{Score}=\text{DEGs}\left(\text{HR}>1\right)*\left(\text{PC}1+\text{PC}2\right)-\text{DEGs}\left(\text{HR}<1\right)*\left(\text{PC}1+\text{PC}2\right). \end{array}$$

The high- and low-score groups were classified based on the median value of the scoring system. The construction of the scoring system was based on the previous study [31].

### 2.4. Ferroptosis-Associated Characteristic in the Scoring System

Shrunken centroids classifier was performed to identify ferroptosis-associated characteristics in the scoring system with *R* package “pamr” as previously reported. The threshold was set as 2.8585337, which was based on the lowest error rate in self-validation.

### 2.5. Potential Mechanisms Prediction

The GO and KEGG analyses were analyzed by conducting the GSVA and the GSEA analysis, respectively [32, 33].

### 2.6. The Immune Landscape Prediction

The immune landscape of ovarian cancer with different subtypes was analyzed with the ESTIMATE algorithm, the 28 immunocytes project, and the 64 × cell analysis as previously described [34].

### 2.7. Drug Prediction

Drug sensitivity data were acquired from the PRISM drug repurposing resource (https://depmap.org/repurposing/) and Cancer Therapeutics Response Portal (CTRP, http://portals.broadinstitute.org/ctrp/) data set. Cell line expression matrix for was downloaded from the Broad-Novartis Cancer Cell Line Encyclopedia (CCLE, https://portals.broadinstitute.org/ccle). The lower AUC value of samples indicated higher sensitivity to this kind of drugs. The AUC value was calculated with *R* package “pRRophetic” as previous work [35].

### 2.8. Statistical Analysis

The student *t*-test and ANOVA test were applied to verify the difference between two comparisons and multiple comparisons, respectively. Kaplan-Meier curves were generated for overall survival analysis, while the Log-rank test was introduced to test the survival analysis. The value *p* < 0.05 was considered significant. All analyses were performed by using *R* (version 3.6.2).

## 3. Results

### 3.1. Ferroptosis Activation May Affect Ovarian Cancer Progression and Invasion Ability

**Figure 1:**
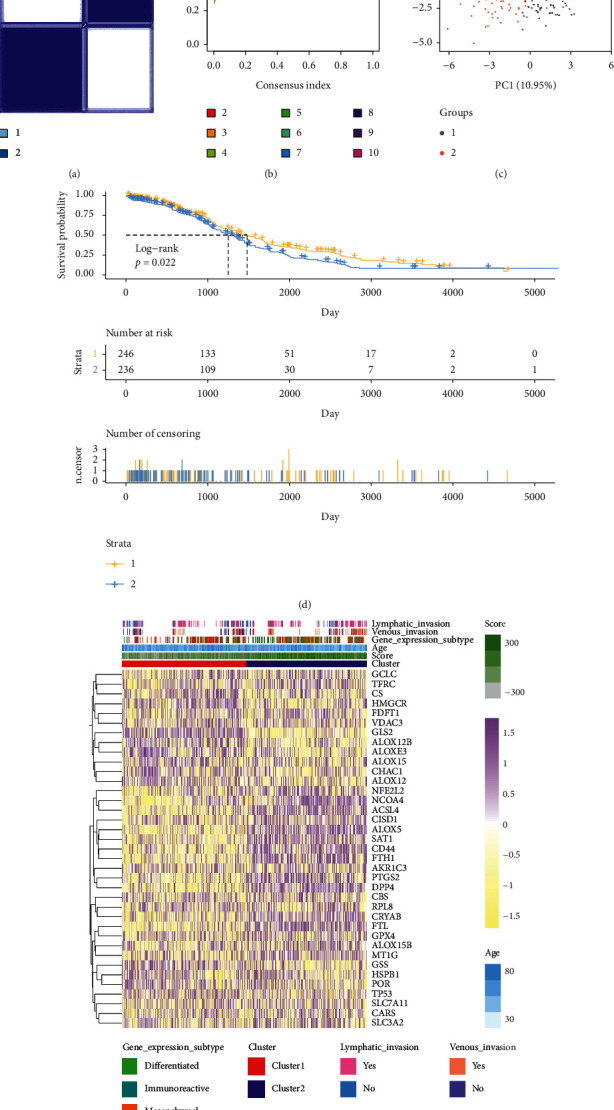
Cluster model of TCGA array-Agilent data set was constructed based on the expression levels of ferroptosis-related genes. The clinical characteristics and prognosis of the two clusters were analyzed. (a and b) Consistent cluster analysis was used to divide ovarian cancer into different clusters, and it was found that the clustering effect of two clusters was the best. (c) The results of PCA visualization analysis also fit into two clusters. (d) Survival analysis showed that the overall survival rate in cluster 2 decreased significantly, *p*=0.022. (e) The heat map showed that the expression of ferroptosis tolerance-related genes increased in cluster 2, and the clinical characteristics were more malignant than that in cluster 1.

According to the expression level of ferroptosis-related genes, ovarian cancer patients in the TCGA array-Agilent training data set were divided into different clusters using the consistent cluster analysis, and the cluster model was constructed (Figures 1(a)–1(c)).

The significant difference in overall survival outcome was identified between clusters 1 and 2. Compared with cluster 1, samples from cluster 2 showed a lower overall survival rate and worse prognosis (*p*=0.022; Figure 1(d)). The results of heat map analysis of TCGA array-Agilent data set showed the expression profiles of ferroptosis-related genes in two clusters. We observed an elevated trend in the expression of ferroptosis tolerance-related genes in cluster 2 (Figure 1(e)), suggesting that ferroptosis affects the prognosis of ovarian cancer. Moreover, samples from cluster 2 also manifested stronger lymph node invasion and venous invasion tendency, implying ferroptosis may affect tumor progression by modulating tumor invasion. Previous studies reported four subtypes of ovarian cancer based on gene expression, including differentiated, immunoreactive, proliferative, and mesenchymal subtypes [6]. The literature showed that mesenchymal had the worst survival outcome and highest invasion ability than other subtypes [7], suggesting its high malignancy. Meanwhile, we identified that the proportion of mesenchymal subtypes in cluster 2 was higher than that in cluster 1, which further supported the conclusion that ferroptosis can affect ovarian cancer progression by regulating tumor invasion. In general, the established cluster model based on ferroptosis-related genes highlights the potential relationship among ferroptosis, tumor invasion, and ovarian cancer progression.

### 3.2. Build a Score Risk Signature Model Based on LASSO Algorithm

**Figure 2:**
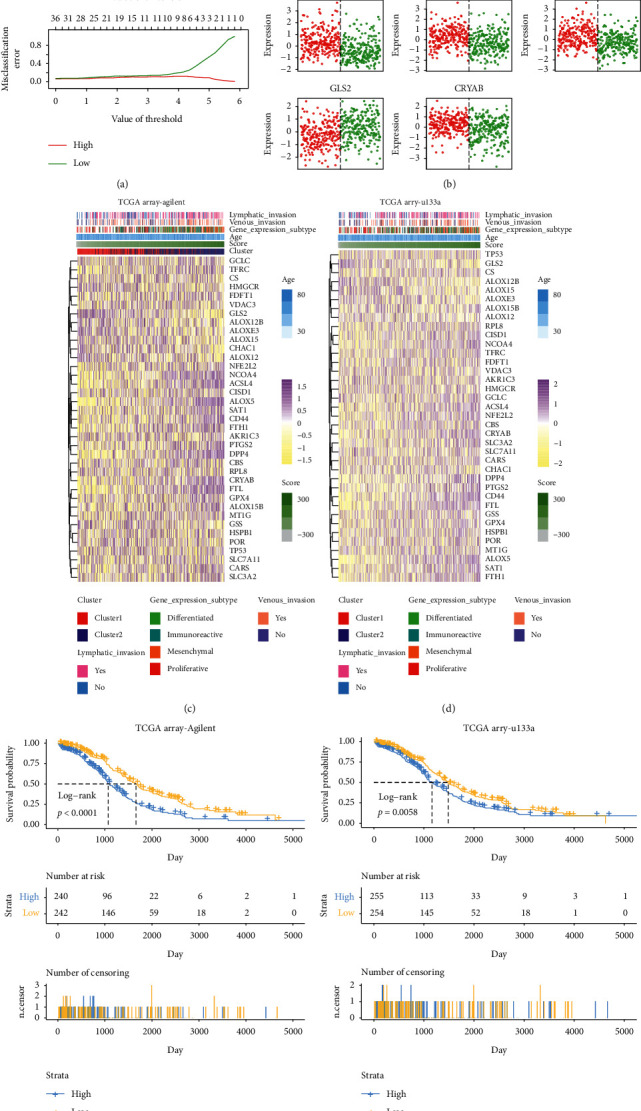
The ferroptosis-related genetic characteristics were identified, and the clinical features and prognosis of the two subgroups were compared. (a) PAMR used the Shrunken Centroids classifier to identify ferroptosis-related genetic characteristics between high- and low-score groups. (b) ferroptosis-related genetic characteristics between high- and low-score groups. (c, d) Heat map analysis of the TCGA array-Agilent data set showed a higher degree of clinical characteristic malignancy in the group with the higher score, and similar results were found in the TCGA array-u133a data set. (e, f) Survive analysis was performed on the TCGA array-Agilent data set (*p* < 0.0001) and TCGA array-u133a data set (*p*=0.0058) based on the score signature prognostic model.

To further quantify the clustering model, we first identified 909 differential expression genes between clusters 1 and 2 (Supplementary Table 1); then univariate Cox regression analysis and LASSO regression algorithm were applied to excavate cancer-progression-associated genes successively in the TCGA array-Agilent training data set. Thirteen genes were filtered out as the potential mechanisms-associated genes between clusters 1 and 2 eventually, including TGM2, ASAH1, STK3, RIN2, HERC3, FTH1, FMO2, EHD2, COL8A2, C5AR1, AXL, ADORA3 and ADAM9, and a score risk signature model was constructed (Supplementary Figures 2(a)–2(c)). The ROC result illustrated that the score signature model was more precise in predicting survival outcome of ovarian cancer patients than the cluster model (Supplementary Figure 3(b)). Then we identified ferroptosis-related genetic characteristics between high- and low-score groups that further strengthened the link (Figures 2(a) and 2(b)). Similarly, the heat map also mapped more lymph node and venous invasion samples and more mesenchymal subtype tumor in the high-score group, which supported the accuracy of the cluster model (Figure 2(c)). Similar results were also obtained in the TCGA array-u133a data set (Figure 2(d)) and TCGA seq data set (Supplementary Figure 3(a)). Survival analysis (Figure 2(e)) showed that samples with higher scores had shorter median survival times than low-score samples both in the training cohort (*p* < 0.0001) and the TCGA array-u133a validation data set (*p*=0.0058, Figure 2(f)). However, in the TCGA seq validation data set, samples with higher scores seemed to have a worse clinical outcome, but there was no significant difference according to the log-rank test (*p*=0.055; Supplementary Figure 3(c)), which may be due to the small sample size of the TCGA seq data set. Notably, ferroptosis tolerance-related genes were also upregulated in the high-score group. In conclusion, the scoring system was not only able to predict ovarian cancer progression but also associated with ferroptosis.

### 3.3. High-Score Samples Are Associated with Malignancy Clinical Characteristics

**Figure 3:**
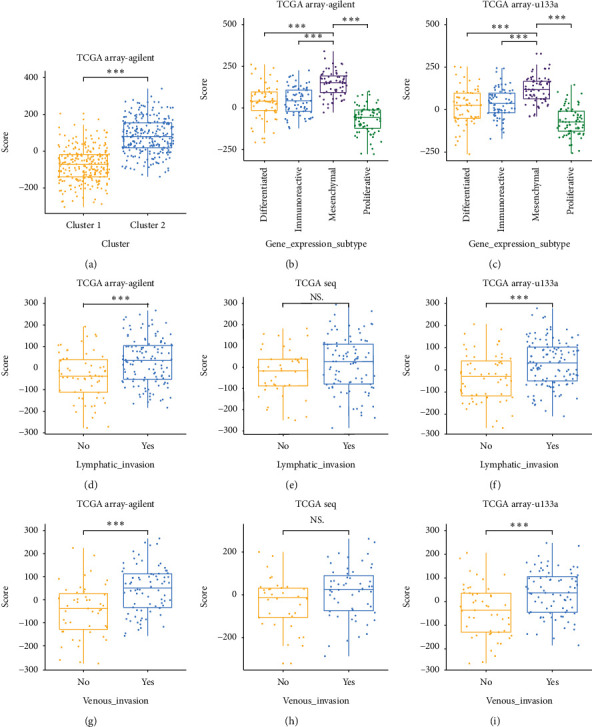
The score of different clinical characteristics subgroups were compared. (a) Score value comparison between two clusters based on cluster model in TCGA array-Agilent data set. (b and c) Score value comparison between four subgroups based on gene expression in TCGA array-Agilent training data set and TCGA array-u133a validation data set. (d–f) Score value comparison between two subgroups with or without lymph node invasion in TCGA array-Agilent training data set, TCGA seq, and TCGA array-u133a validation data set. (g–i) Score value comparison between two subgroups with or without venous invasion in TCGA array-Agilent training data set, TCGA seq, and TCGA array-u133a validation data set. NS: no significance, ^*∗*^*p* < 0.05, ^*∗∗*^*p* < 0.01, and ^*∗∗∗*^*p* < 0.001.

As illustrated, high-score samples mostly belonged to cluster 2 (Figure 3(a)), which indicated the potential relationship between the cluster model and the scoring system. Moreover, high-score samples were more likely to be classified as mesenchymal subtype, the more malignant subtype than other three subtypes, such as differentiated, immunoreactive, and proliferative subtypes (Figures 3(b) and 3(c)).

Critically, the average score of samples in the lymph node invasive tumors is higher than non-invasive tumors (Figure 3(d)). Similar results were obtained in the validation cohorts (Figure 3(f)). The venous invasive tumors also had a higher score than non-invasive tumors (Figures 3(g) and 3(i)). In the TCGA seq data set, the average score had no significant difference between invasive and non-invasive tumors (Figure 3(e)), although more high-score samples were enriched in invasive tumor groups than non-invasive tumor groups (Figure 3(h)). This contradictory result may result from the number of samples in the TCGA seq data set. To sum up, high-score samples were associated with malignant clinical characteristics.

### 3.4. High-Score Group May Affect Tumor Progression through Regulating Tumor Invasion and Immune Infiltration

**Figure 4:**
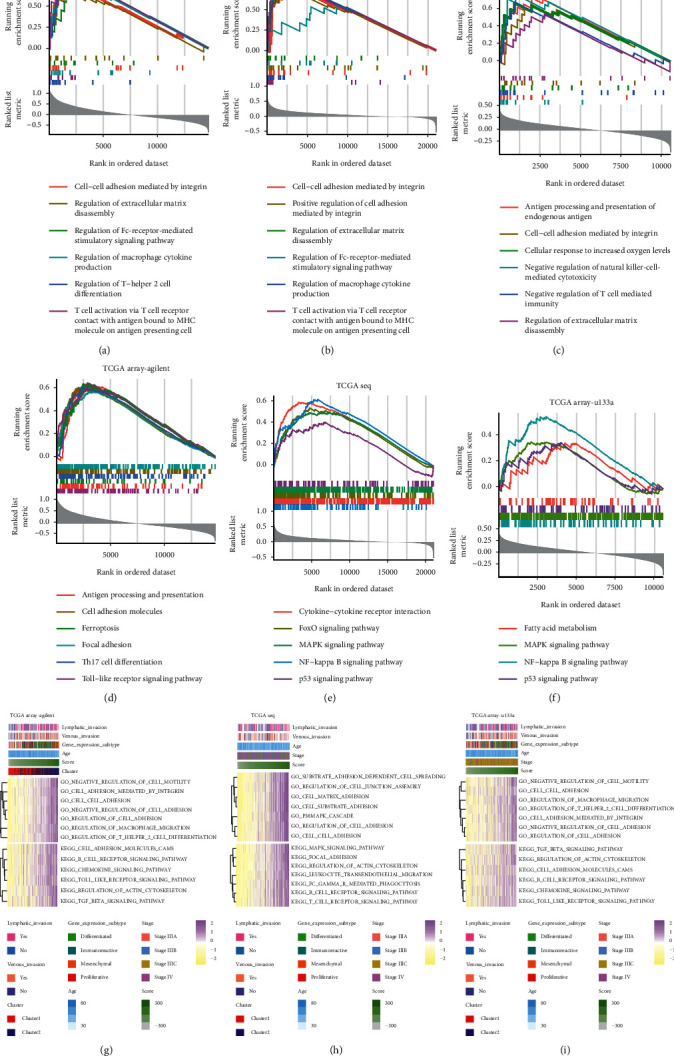
The results of Go and KEGG analyses based on GSEA and GEVA analyses were used for functional studies. (a–c) The result of GO analysis based on GSEA analysis. (d–f) The result of KEGG analysis based on GSEA analysis. (g–i) The results of GO and KEGG analyses based on GSVA analysis.

In order to explore the potential mechanism of how ferroptosis activation affects tumor progression, we used the scoring system for subsequent analysis. GO and KEGG enrichment analyses based on GSEA and GSVA analyses were performed. In the training cohort, GO analysis results based on GSEA analysis showed that pathways like cell-cell adhesion mediated by integrin and regulation of extracellular matrix disassembly were activated in the high-score group. Besides, immunocytes infiltration was also different between high- and low-score groups. Immune invasion-related pathways including regulation of macrophage cytokine production, regulation of T-helper 2 cell differentiation, and other biological pathways (Figure 4(a)) were also enriched in the high-score group. A similar conclusion can also be obtained from the KEGG analysis (Figure 3(d)). Therefore, high-score samples may promote tumor progression by regulating tumor invasion ability and immune infiltration. Moreover, the results of GO and KEGG enrichment analyses based on the GSVA algorithm also suggested high-score samples promoted tumor invasion through activating multiple pathways, including negative regulation of cell motility, cell adhesion mediated by integrin, regulation of macrophage migration, regulation of T-helper 2 cell differentiation, TGF-*β* signaling pathway, and so on (Figure 4(g)). Similar results were obtained in the validation data sets (Figures 4(b), 4(c), 4(e), 4(f), 4(h), and 4(i)). In conclusion, the high-score group promoted tumor progression by affecting tumor invasion ability and immune infiltration.

### 3.5. The Immune Infiltration of High-Score Group

**Figure 5:**
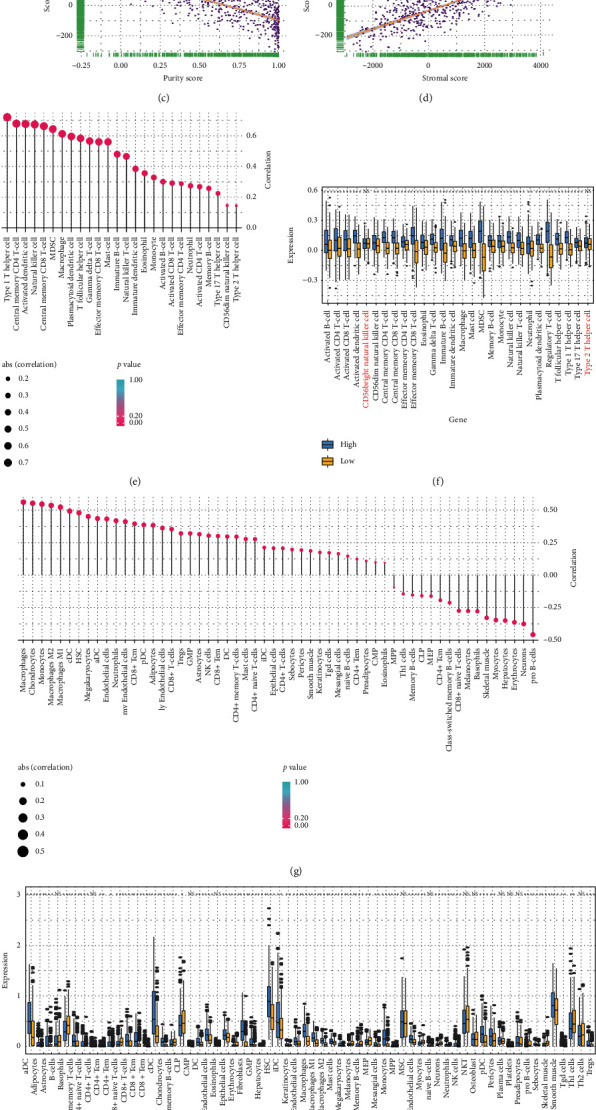
Immune infiltration analysis based on score risk signature in TCGA array-Agilent data set. (a) Estimate score was positively correlated with score (correlation coefficient *r* = 0.75and *p*=0). (b) Immune score (correlation coefficient *r* = 0.6 and *p*=0) was positively correlated with score. (c) Stromal score (correlation coefficient *r* = 0.8 and *p*=0) was positively correlated with score. (d) Purity score (correlation coefficient *r* = -0.73 and*p*=0) was negatively correlated with score. (e and f) The correlation between 28 immune cells and score signature and their expression levels. (g and h) The correlation and expression levels of 64 cells (including immune cells and stromal cells) with score signature. NS: no significance, ^*∗*^*p* < 0.05, ^*∗∗*^*p* < 0.01, and ^*∗∗∗*^*p* < 0.001.

Immunocytes such as invasive immune cells and stromal cells infiltrating the tumor microenvironment are thought to play an important role in tumor growth, progression, and drug resistance [33, 34]. In the TCGA array-Agilent training data set, we used the estimate algorithm to calculate estimate score, immune score, purity score, and stromal score to explain immune cell and stromal cell infiltration situation. The score prognostic model was positively correlated with estimate score (correlation coefficient *r* = 0.75 and *p*=0; Figure 5(a)), immune score (correlation coefficient *r* = 0.6 and *p*=0; Figure 5(b)), and stromal score (correlation coefficient *r* = 0.8 and *p*=0; Figure 5(d)). Correspondingly, the negative correlation between the purity score and the score prognostic model (correlation coefficient *r* = −0.73 and *p*=0; Figure 5(c)) indicated that there was a difference in immune infiltration between the high- and the low-score groups. The same tendency was explored both in TCGA seq and TCGA array-u133a data sets.

Then we analyzed the distribution proportion of 28 kinds of immune cells and 64 kinds of cells (including immune cells and stromal cells). Results of 28 kinds of immune cells analysis showed that the activated CD4 T cell, activated CD8 T cell, regulatory T cells, and macrophage were positively correlated with score (Figure 5(e)). In the meantime, those cells were statistically increased in the high-score group (Figure 5(f)). The analysis results of 64 kinds of cells showed that macrophages, CD8+ Tcm, NK cell, dendritic cells, regulatory T cell (Tregs), adipocytes, endothelial cells, epithelial cells, keratinocytes, fibroblast, and other non-tumor cells were positively correlated with scores (Figure 5(g)) while Pro B cells, CD8+ naive T cells, CD4+ Tcm, Erythrocytes, basophils, and so on were negatively correlated with score. Except for B cells, CD4+ T cells, CMP, MSC, naive B cells, NKT, and other 11 types of cells, the remaining cells were statistically different between high- and low-score groups (Figure 5(h)). Similar results were also identified in TCGA seq (Supplementary Figures 4(a)–4(h)) and TCGA array-u133a (Supplementary Figures 5(a)–5(h)) validation data sets. The analysis results of 28 kinds of immune cells and 64 kinds of cells showed that macrophages and regulatory T cells were statistically elevated in the high-score group. According to our previous results, the high-score group had lower overall survival and more lymph node and venous invasion. A previous study reported that tumor-associated macrophages (TAMs), and regulatory T cells (Tregs) facilitated ovarian cancer immune surveillance evasive and tumor metastasis [36], which is consistent with our results. Taken together, these results suggest that samples with high expression of ferroptosis-related genes can recruit multiple immune cells and stromal cells in the tumor microenvironment to facilitate tumor invasive and metastasis.

### 3.6. Identification of Potential Targeted Drugs to High-Score Group

**Figure 6:**
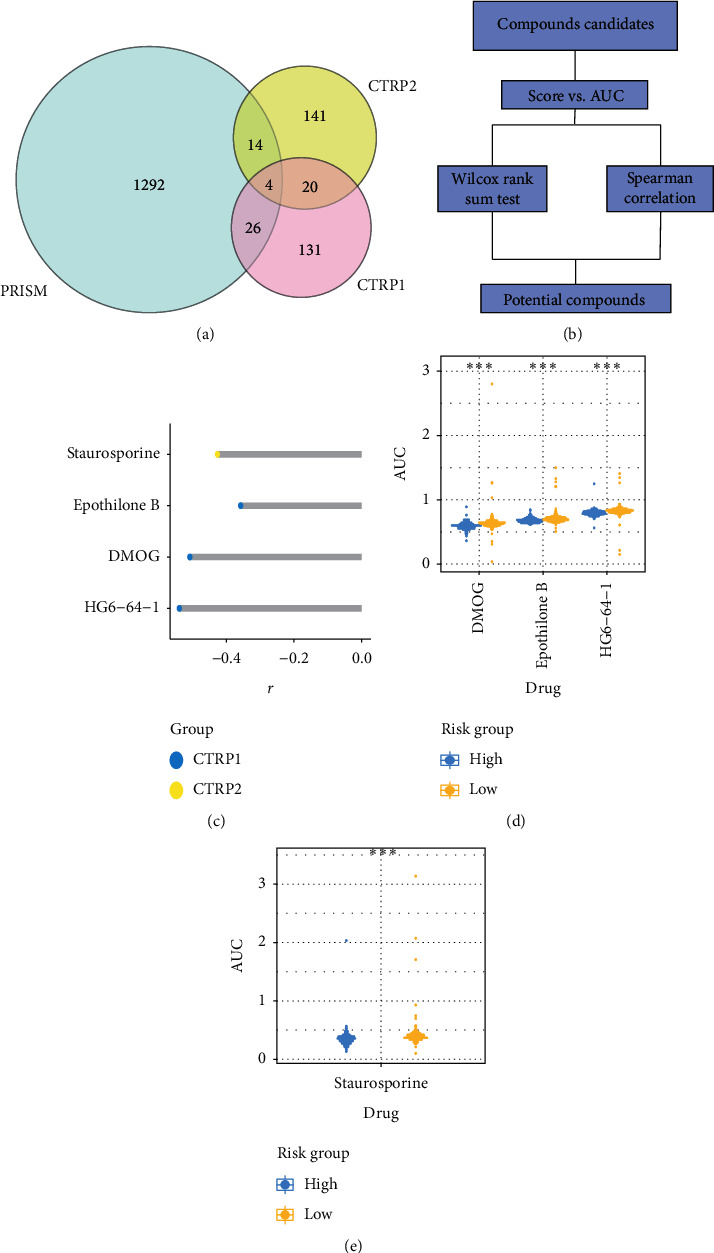
Clinical drug prediction. (a–c) Drug selection process. (d) The relationship between AUC and score signature of 3 drugs from CTRP1. (e) The relationship between AUC and score signature for 1 drug of CTRP2. NS: no significance, ^*∗*^*p* < 0.05, ^*∗∗*^*p* < 0.01, and ^*∗∗∗*^*p* < 0.001.

Considering that the score signature can predict the prognosis of ovarian cancer and it is related to tumor invasion, then we predicted potential drugs for high-score group. Three drug data sets, Prism, CTRP1, and CTRP2, were collected and used to predict potential sensitive drugs (Figure 6(a)). The AUC value of each drug to each sample was calculated, and a lower AUC value indicated higher sensitivity. The selection criteria of potential sensitive drugs were that drugs should meet both statistical difference in AUC value between high- and low-score groups and the correlation coefficient *r* of Spearman correlation was greater than 0.3 (Figure 6(b)). Finally, four potential sensitive drugs, including staurosporine, epothilone B, DMOG, and HG6-64-1, were screened out (Figures 6(c)–6(e)). It has been previously reported that staurosporine [37], epothilone B [38, 39], and HG6-64-1 have a therapeutic effect on ovarian cancer [40]. Critically, we identified that a novel potential sensitive drug is dimethyloxallyl glycine (DMOG), a kind of prolyl hydroxylase inhibitor (PHI), may be able to treat invasive ovarian cancer. In terms of the association between DMOG and lipid metabolism, studies have found that DMOG can increase lipid accumulation in human primary renal tubular epithelial cells [41] and lipid absorption in macrophages [42]. Therefore, DMOG may serve as a novel potential targeted drug for ovarian cancer.

## 4. Discussion

In this work, we first analyzed the subtype of ovarian cancer based on ferroptosis-related genes expression. Two clusters, clusters 1 and 2, were further identified. Meanwhile, higher ferroptosis-resistant-associated genes were noticed in cluster 2. Moreover, the median survival time of cluster 2 was shorter than cluster 1. Previous studies reported that MAP30 can prevent ovarian cancer progression through modulating ferroptosis [43]. Superparamagnetic iron oxide nanoparticles can induce ferroptosis to inhibit the progression of ovarian cancer stem cells [44]. Therefore, the sensitivity of ovarian cancer cells to ferroptosis may affect tumor progression [22].

In order to further depict the difference between clusters 1 and 2, the scoring system was constructed. As illustrated, samples from cluster 2 were more likely been calculated with a higher score. Interestingly, higher ferroptosis-resistant genes expression and worse tumor prognosis were also noticed in high-score samples. Critically, high-score samples also showed aggressive growth patterns, including venous invasion and lymph node invasion suggesting ferroptosis may affect ovarian cancer progression by modulating tumor invasion ability. The potential mechanisms analysis also supported that more tumor metastasis-associated pathways were activated in high-score samples. Previous studies also proved that ferroptosis can affect tumor migration and invasion including colorectal cancer [45], melanoma [46], breast cancer [47], prostate tumor [48], and so on. Collectively, this work proposed that the activation of ferroptosis may affect ovarian cancer progression by modulating tumor metastasis.

The immune landscape was further depicted between high- and low-score samples. Immune score and stromal score were positively correlated with the scoring system. Besides, the infiltration ratio of tumor progression-associated immunocytes such as macrophages, CD8+ Tcm, NK cell, and dendritic cells regulatory T cell (Tregs) were upregulated in high-score samples. Previous study suggested that higher stromal signature and M2 macrophages can affect ovarian cancer sensitivity to immunotherapy [49]. Cisplatin can facilitate ovarian cancer migration by stimulating macrophage [50]. The infiltration ratio of immunocytes can also modulate tumor response to immunotherapy [51, 52]. Moreover, ferroptosis can also regulate tumor immune landscape and tumor response to chemotherapy [28, 53–55]. Therefore, ferroptosis can also affect tumor immune landscape by regulating tumor progression.

Four potential drugs were identified according to the scoring system, including staurosporine, epothilone B, DMOG, and HG6-64-1. Critically, DMOG was identified as novel potential targeted drugs for ovarian cancer. DMOG induced chemical hypoxia and stabilized hypoxia-inducible factor-1 alpha. Hypoxia is associated with increased resistance to chemotherapy and poor overall prognosis in many cancers [56]. Hypoxia has been reported to promote the proliferation, migration, and invasion in prostate cancer [57]. In glioblastoma, hypoxia-inducible factor 1*α* inhibited the expression of tryptophan-2, 3-dioxygenase and modulates antitumor immunity [58]. Meanwhile, loss of hypoxia-inducible factor-1 in malignant epithelial cells and myeloid cells limited tumor growth [59, 60]. All these studies indicated that hypoxia and hypoxic-inducing factors promote tumor progression and metastasis. Another research reported that DMOG can significantly reduce tumor metastasis by inactivating fibroblasts in liver and lung [61]. However, the relationship between DMOG and ovarian cancer is still elusive and require further exploration.

## 5. Conclusions

In summary, we constructed a scoring system in ovarian cancer based on the expression of ferroptosis-related genes. This scoring system was not only able to predict tumor progression but also suggest the potential association among ferroptosis, ovarian cancer, and tumor metastasis. Besides, we also verified that ferroptosis can also modulate the component of tumor immune landscape. Critically, DMOG was identified as a novel potential drug to treat ovarian cancer progression.

## Data Availability

All the data analyzed in this work were downloaded from TCGA (https://xenabrowser.net/).

## Supplementary Materials

Supplementary Materials Supplementary Figure 1: the workflow of this article. Supplementary Figure 2: univariate Cox regression and LASSO regression were used to establish a risk score model containing 13 ferroptosis-related genes. Supplementary Figure 3: heat map and survival analysis of TCGA seq data set and ROC curve of TCGA array-Agilent data set. Supplementary Figure 4: immune infiltration analysis based on score signature in TCGA seq data set. Supplementary Figure 5: immune infiltration analysis based on score signature in TCGA array-u133a data set. Supplementary Table1: Cancer-progression-associated genes were successively excavated in the TCGA array-Agilent training data set.
