# Supplementary material for: Ferroptosis-Related Gene Signature Promotes Ovarian Cancer by Influencing Immune Infiltration and Invasion
Source: J Oncol. 2021 May 26;2021:9915312. doi: 10.1155/2021/9915312 (PMC8175133; doi:10.1155/2021/9915312)
Supplement: Supplementary Materials — Supplementary Figure 1: the workflow of this article. Supplementary Figure 2: univariate Cox regression and LASSO regression were used to establish a risk score model containing 13 ferroptosis-related genes. Supplementary Figure 3: heat map and survival analysis of TCGA seq data set and ROC curve of TCGA array-Agilent data set. Supplementary Figure 4: immune infiltration analysis based on score signature in TCGA seq data set. Supplementary Figure 5: immune infiltration analysis based on score signature in TCGA array-u133a data set. Supplementary Table1: Cancer-progression-associated genes were successively excavated in the TCGA array-Agilent training data set. [file 9915312.f1.zip › 9915312.f1/Supplementary Materials legends revised.docx]

**Supplementary Figure 1.** The workflow of this article.

**Supplementary Figure 2.** The risk score model was established. (A-C) Univariate cox regression and LASSO regression were used to establish a risk score model containing 13 ferroptosis-related genes.

**Supplementary Figure 3.** Heat map and survival analysis of TCGA seq dataset and ROC curve of TCGA array-Agilent dataset. (A**)** Heat map analysis of the TCGA seq dataset showed the clinical features are more malignant in the group with higher score. (B) ROC curve result illustrated that the score signature model is more precise in predicting ovarian cancer patents survival outcome than the cluster model. (C) Survival analysis result showed that the overall survival rate in cluster 2 decreased, but had no significance, *p*=0.055.

**Supplementary Figure 4.** Immune infiltration analysis based on score signature in TCGA seq dataset. (A) Estimate score was positively correlated with score (correlation coefficient r=0.65, *p*=0). (B) Immune score was positively correlated with score (correlation coefficient r=0.48, *p*=0). (C) Stromal score (correlation coefficient r=0.72, *p*=0) was positively correlated with score. (D) Purity score was negatively correlated with score (correlation coefficient r=-0.65, *p*=0). (E, F) The correlation between 28 immune cells and score signature and their expression levels were different. (G, H) The correlation and expression levels of 64 cells (including immune cells and stromal cells) with score signature and expression levels of most cells were different. NS: no significance, *: *p*< 0.05, **: *p* < 0.01, *** means *p* <0.001.

**Supplementary Figure 5.** Immune infiltration analysis based on score signature in TCGA array-u133a dataset. (A) Estimate score was positively correlated with score (correlation coefficient r=0.64, *p*=0). (B)Immune score (correlation coefficient r=0.55, *p*=0) was positively correlated with score. (C) Stromal score (correlation coefficient r=0.65, *p*=0) was positively correlated with score. (D) Purity score (correlation coefficient r=-0.62, *p*=0) was negatively correlated with score. (E, F) The correlation between 28 immune cells and score signature and their expression levels were different. (G, H) The correlation and expression levels of 64 cells (including immune cells and stromal cells) with Score signature and expression levels of most cells were different. NS: no significance, *: *p*< 0.05, **: *p* < 0.01, *** means *p* <0.001.

**Supplementary Table1.** Univariate Cox regression analysis and LASSO regression algorithm were applied to excavated cancer progression associated genes successively in the TCGA array-Agilent training dataset.
