# Supplementary material for: Ferroptosis-Related Gene Signature Promotes Ovarian Cancer by Influencing Immune Infiltration and Invasion
Source: J Oncol. 2021 May 26;2021:9915312. doi: 10.1155/2021/9915312 (PMC8175133; doi:10.1155/2021/9915312)
Supplement: Supplementary Materials — Supplementary Figure 1: the workflow of this article. Supplementary Figure 2: univariate Cox regression and LASSO regression were used to establish a risk score model containing 13 ferroptosis-related genes. Supplementary Figure 3: heat map and survival analysis of TCGA seq data set and ROC curve of TCGA array-Agilent data set. Supplementary Figure 4: immune infiltration analysis based on score signature in TCGA seq data set. Supplementary Figure 5: immune infiltration analysis based on score signature in TCGA array-u133a data set. Supplementary Table1: Cancer-progression-associated genes were successively excavated in the TCGA array-Agilent training data set. [file 9915312.f1.zip › 9915312.f1/Supplementary table 1 (1).docx]

| Gene | HR | HR.95L | HR.95H | pvalue |
| --- | --- | --- | --- | --- |
| EVI2A | 1.138489 | 1.015795 | 1.276004 | 0.025793 |
| TLR4 | 1.156551 | 1.033182 | 1.29465 | 0.011499 |
| VCAM1 | 1.06245 | 0.948819 | 1.189689 | 0.293882 |
| TGFBI | 1.218959 | 1.080173 | 1.375578 | 0.001325 |
| NNMT | 1.12151 | 0.996159 | 1.262635 | 0.057917 |
| PLAU | 1.070115 | 0.95477 | 1.199395 | 0.2442 |
| EBI2 | 1.148 | 1.025404 | 1.285254 | 0.016605 |
| C3AR1 | 1.238294 | 1.101876 | 1.391601 | 0.000332 |
| CLEC2B | 1.146687 | 1.0248 | 1.283071 | 0.016976 |
| ITGB2 | 1.087653 | 0.969819 | 1.219803 | 0.15096 |
| CXorf21 | 1.1652 | 1.043969 | 1.30051 | 0.00638 |
| TLR7 | 1.169092 | 1.045939 | 1.306745 | 0.005945 |
| RAB31 | 1.197151 | 1.062575 | 1.348771 | 0.003101 |
| ZEB2 | 1.109577 | 0.991031 | 1.242303 | 0.071281 |
| HCK | 1.133034 | 1.013658 | 1.266468 | 0.027894 |
| TAGAP | 1.082089 | 0.966835 | 1.211082 | 0.169751 |
| DCN | 1.191098 | 1.058619 | 1.340156 | 0.003651 |
| CD53 | 1.08224 | 0.966757 | 1.211518 | 0.169833 |
| LAIR1 | 1.125707 | 1.001255 | 1.265627 | 0.047597 |
| ALOX5AP | 1.206665 | 1.072617 | 1.357466 | 0.001768 |
| PTPRC | 1.047777 | 0.93775 | 1.170713 | 0.40965 |
| NLRC4 | 1.159361 | 1.037256 | 1.295841 | 0.00921 |
| ARHGDIB | 1.037348 | 0.925975 | 1.162116 | 0.526886 |
| LCP2 | 1.063981 | 0.950576 | 1.190915 | 0.280808 |
| RGS18 | 1.154583 | 1.034911 | 1.288093 | 0.010035 |
| F13A1 | 1.169705 | 1.047946 | 1.305612 | 0.00519 |
| MNDA | 1.130679 | 1.008283 | 1.267934 | 0.035634 |
| ITGA4 | 1.132771 | 1.013342 | 1.266275 | 0.028299 |
| FCGR2B | 1.171093 | 1.042455 | 1.315604 | 0.007806 |
| MS4A6A | 1.17802 | 1.051418 | 1.319866 | 0.004738 |
| IL7R | 0.984676 | 0.879276 | 1.102711 | 0.789208 |
| SPP1 | 1.235702 | 1.091838 | 1.398523 | 0.000805 |
| MS4A4A | 1.195163 | 1.064666 | 1.341656 | 0.00251 |
| WISP1 | 1.198447 | 1.064248 | 1.349567 | 0.002811 |
| TLR6 | 1.07996 | 0.96248 | 1.211781 | 0.190487 |
| ATP8B4 | 1.215437 | 1.084511 | 1.362168 | 0.000793 |
| GPR34 | 1.27161 | 1.139843 | 1.418609 | 1.67E-05 |
| GIMAP6 | 1.129352 | 1.009727 | 1.26315 | 0.03322 |
| GPR65 | 1.173686 | 1.05143 | 1.310158 | 0.004323 |
| FLI1 | 1.111275 | 0.993985 | 1.242405 | 0.063745 |
| LILRB3 | 1.089645 | 0.972544 | 1.220845 | 0.138868 |
| FPRL2 | 1.144921 | 1.022014 | 1.282609 | 0.019502 |
| TLR2 | 1.166728 | 1.033229 | 1.317477 | 0.012875 |
| LAPTM5 | 1.110248 | 0.988147 | 1.247436 | 0.078513 |
| C4orf18 | 1.159455 | 1.035712 | 1.297982 | 0.01019 |
| PLAUR | 1.220466 | 1.084469 | 1.373517 | 0.000949 |
| CD33 | 1.128456 | 1.005801 | 1.266068 | 0.039543 |
| AIF1 | 1.085433 | 0.969074 | 1.215764 | 0.156488 |
| CLEC4A | 1.132324 | 1.009128 | 1.27056 | 0.034465 |
| SAMSN1 | 1.082822 | 0.966211 | 1.213508 | 0.171092 |
| PLEK | 1.061072 | 0.948928 | 1.18647 | 0.298274 |
| ARHGEF6 | 1.028034 | 0.913111 | 1.157421 | 0.647589 |
| SLCO2B1 | 1.145003 | 1.019007 | 1.286578 | 0.022815 |
| GIMAP4 | 1.115294 | 0.99846 | 1.2458 | 0.053277 |
| FCGR2A | 1.228511 | 1.088508 | 1.38652 | 0.000857 |
| THBS2 | 1.131441 | 1.008422 | 1.269467 | 0.035485 |
| LUM | 1.14305 | 1.016887 | 1.284867 | 0.025052 |
| CD84 | 1.130786 | 1.011264 | 1.264435 | 0.031046 |
| DPYD | 1.075342 | 0.961635 | 1.202494 | 0.202699 |
| HAVCR2 | 1.083012 | 0.965532 | 1.214785 | 0.17344 |
| ECM2 | 1.152566 | 1.024417 | 1.296746 | 0.01822 |
| MSR1 | 1.237355 | 1.104966 | 1.385606 | 0.000225 |
| DAB2 | 1.180552 | 1.048673 | 1.329016 | 0.006027 |
| GIMAP1 | 1.090436 | 0.976127 | 1.218132 | 0.125445 |
| GPNMB | 1.056508 | 0.945435 | 1.180629 | 0.332087 |
| TFEC | 1.122846 | 1.004891 | 1.254647 | 0.040744 |
| CLEC5A | 1.235468 | 1.100853 | 1.386544 | 0.000328 |
| LY96 | 1.092756 | 0.974503 | 1.225358 | 0.12902 |
| ARHGAP15 | 1.068961 | 0.956266 | 1.194936 | 0.240709 |
| IL1B | 1.163812 | 1.036633 | 1.306594 | 0.01019 |
| OLR1 | 1.153578 | 1.02731 | 1.295366 | 0.015714 |
| TLR8 | 1.166315 | 1.044295 | 1.302593 | 0.006359 |
| CTSK | 1.112356 | 0.992759 | 1.246361 | 0.066545 |
| FAS | 1.185463 | 1.058696 | 1.327407 | 0.003194 |
| GMFG | 1.061116 | 0.947983 | 1.187751 | 0.302406 |
| SRGN | 1.159422 | 1.03021 | 1.304841 | 0.014141 |
| FGF7 | 1.195261 | 1.065801 | 1.340447 | 0.002292 |
| TLR1 | 1.121698 | 0.999368 | 1.259002 | 0.051268 |
| CD14 | 1.143593 | 1.018666 | 1.283841 | 0.023008 |
| ANTXR2 | 1.10486 | 0.980756 | 1.244668 | 0.100938 |
| C2orf32 | 1.187071 | 1.055362 | 1.335217 | 0.004264 |
| KIAA1913 | 1.13575 | 1.01623 | 1.269327 | 0.024849 |
| CYBB | 1.07405 | 0.959609 | 1.202139 | 0.213969 |
| NT5E | 1.150802 | 1.028439 | 1.287724 | 0.014331 |
| ALOX5 | 1.113309 | 0.987327 | 1.255367 | 0.079808 |
| EVI2B | 1.085316 | 0.972852 | 1.210781 | 0.142417 |
| PSCD4 | 1.079992 | 0.963057 | 1.211126 | 0.188121 |
| NCF2 | 1.057997 | 0.942947 | 1.187083 | 0.337145 |
| FAP | 1.142959 | 1.015276 | 1.286699 | 0.027049 |
| GPR84 | 1.137355 | 1.015798 | 1.273459 | 0.02563 |
| OLFML2B | 1.129648 | 1.011711 | 1.261333 | 0.030241 |
| RAB8B | 1.126803 | 0.997725 | 1.272579 | 0.054446 |
| CXorf9 | 1.04026 | 0.928663 | 1.165268 | 0.49542 |
| FIBIN | 1.192056 | 1.067328 | 1.33136 | 0.001837 |
| SERPINF1 | 1.11062 | 0.988386 | 1.24797 | 0.077798 |
| NPL | 1.120888 | 0.999208 | 1.257385 | 0.051601 |
| SLAMF8 | 1.108432 | 0.990143 | 1.240853 | 0.073788 |
| EPB41L3 | 1.154174 | 1.024179 | 1.300668 | 0.018681 |
| FYB | 1.049727 | 0.937377 | 1.175544 | 0.400763 |
| BTK | 1.042128 | 0.929984 | 1.167796 | 0.477475 |
| FILIP1L | 1.115792 | 0.992464 | 1.254445 | 0.066745 |
| SMPDL3A | 1.094686 | 0.97698 | 1.226572 | 0.119067 |
| LXN | 1.079391 | 0.959277 | 1.214545 | 0.204354 |
| HOM-TES-103 | 1.045686 | 0.931871 | 1.173401 | 0.447357 |
| MFSD1 | 1.146863 | 1.019238 | 1.290467 | 0.022813 |
| ARHGAP18 | 1.135557 | 1.019511 | 1.264812 | 0.020817 |
| APBB1IP | 1.071549 | 0.955506 | 1.201685 | 0.237333 |
| IGFL2 | 1.076017 | 0.959226 | 1.207027 | 0.211363 |
| NCF4 | 1.123536 | 1.004755 | 1.25636 | 0.041036 |
| C1S | 1.041948 | 0.932489 | 1.164255 | 0.468057 |
| IGSF6 | 1.143954 | 1.025056 | 1.276645 | 0.016309 |
| FGL2 | 1.137627 | 1.017149 | 1.272376 | 0.023966 |
| SIGLEC9 | 1.143532 | 1.020665 | 1.281191 | 0.020742 |
| BHLHB5 | 1.100338 | 0.979076 | 1.236618 | 0.108491 |
| IL10RA | 1.096103 | 0.979355 | 1.226769 | 0.110285 |
| RNASE2 | 1.234085 | 1.094745 | 1.39116 | 0.00058 |
| GNG2 | 1.160516 | 1.029157 | 1.308642 | 0.015145 |
| FN1 | 1.139427 | 1.01309 | 1.281519 | 0.029491 |
| ITGAM | 1.202028 | 1.067173 | 1.353924 | 0.002439 |
| ALDH1A3 | 1.177031 | 1.04563 | 1.324945 | 0.00696 |
| CLEC7A | 1.109795 | 0.99238 | 1.241103 | 0.067867 |
| STX11 | 1.084256 | 0.967538 | 1.215054 | 0.1639 |
| GIMAP8 | 1.081781 | 0.964284 | 1.213595 | 0.180248 |
| CD48 | 1.039313 | 0.931903 | 1.159102 | 0.488431 |
| CPA3 | 1.118986 | 0.999278 | 1.253035 | 0.051481 |
| AMICA1 | 1.003794 | 0.89572 | 1.124908 | 0.948051 |
| PMP22 | 1.169017 | 1.045738 | 1.306827 | 0.006023 |
| GLRX | 1.101857 | 0.983572 | 1.234369 | 0.094117 |
| MAF | 1.201252 | 1.070261 | 1.348276 | 0.001854 |
| VAMP5 | 1.075788 | 0.958137 | 1.207886 | 0.21636 |
| CD300LF | 1.097402 | 0.981577 | 1.226896 | 0.102424 |
| PTGDR | 1.077786 | 0.966481 | 1.201909 | 0.178003 |
| C1orf162 | 1.137324 | 1.015313 | 1.273997 | 0.026254 |
| CHSY-2 | 1.093245 | 0.973762 | 1.227388 | 0.131117 |
| TNFAIP6 | 1.117714 | 0.991443 | 1.260068 | 0.068842 |
| AOAH | 1.120564 | 1.003501 | 1.251283 | 0.043172 |
| LAT2 | 1.091062 | 0.972495 | 1.224085 | 0.137598 |
| COL3A1 | 1.157285 | 1.032259 | 1.297454 | 0.012271 |
| PRDM1 | 1.021419 | 0.916961 | 1.137778 | 0.700219 |
| MS4A7 | 1.227416 | 1.096166 | 1.374381 | 0.000383 |
| LILRB1 | 1.100612 | 0.98221 | 1.233287 | 0.098768 |
| DOK2 | 1.14287 | 1.018493 | 1.282435 | 0.023107 |
| NLRP3 | 1.121803 | 0.998285 | 1.260603 | 0.053468 |
| GJB2 | 1.144237 | 1.017385 | 1.286907 | 0.024611 |
| RGS4 | 1.173535 | 1.043896 | 1.319273 | 0.007379 |
| DSE | 1.161186 | 1.034172 | 1.3038 | 0.011456 |
| FPR1 | 1.153846 | 1.028253 | 1.294778 | 0.01494 |
| MMP2 | 1.116528 | 0.99205 | 1.256625 | 0.067607 |
| SLA | 1.037109 | 0.925699 | 1.161928 | 0.52973 |
| FTHP1 | 1.10501 | 0.986898 | 1.237257 | 0.083398 |
| 1-Mar | 1.186198 | 1.061971 | 1.324958 | 0.002485 |
| TNFSF13B | 1.062217 | 0.948365 | 1.189737 | 0.296738 |
| RNASE6 | 1.148544 | 1.022213 | 1.290487 | 0.019833 |
| SAMD3 | 1.045936 | 0.932777 | 1.172824 | 0.442026 |
| SPARC | 1.132788 | 1.009282 | 1.271406 | 0.034274 |
| CCR5 | 1.104559 | 0.988536 | 1.234198 | 0.079032 |
| LY86 | 1.12737 | 1.003792 | 1.266161 | 0.042985 |
| SLC9A9 | 1.093394 | 0.975583 | 1.225433 | 0.124788 |
| LPPR4 | 1.065134 | 0.951409 | 1.192452 | 0.273378 |
| SNAI2 | 1.11795 | 0.997123 | 1.253417 | 0.056056 |
| THBS1 | 1.14467 | 1.020486 | 1.283966 | 0.021106 |
| WDFY4 | 1.044938 | 0.937091 | 1.165198 | 0.428999 |
| VCAN | 1.089877 | 0.969555 | 1.225132 | 0.149317 |
| COL5A2 | 1.145846 | 1.020116 | 1.287072 | 0.021686 |
| PTPN22 | 1.04892 | 0.933817 | 1.17821 | 0.420623 |
| C5orf29 | 1.198735 | 1.074639 | 1.337161 | 0.00115 |
| CFH | 1.083148 | 0.965428 | 1.215222 | 0.173638 |
| TNFAIP8L2 | 1.073235 | 0.962746 | 1.196403 | 0.202291 |
| GFPT2 | 1.202068 | 1.072958 | 1.346714 | 0.0015 |
| GAB3 | 1.042382 | 0.928702 | 1.169977 | 0.481111 |
| ITGA1 | 1.073312 | 0.961553 | 1.198061 | 0.207265 |
| CST7 | 1.020668 | 0.907677 | 1.147725 | 0.732533 |
| KLRB1 | 1.04911 | 0.939248 | 1.171822 | 0.395629 |
| CRTAM | 1.083584 | 0.972678 | 1.207136 | 0.145083 |
| NCKAP1L | 1.033564 | 0.923398 | 1.156874 | 0.565908 |
| PAPSS2 | 1.090516 | 0.970561 | 1.225296 | 0.145008 |
| LRRC25 | 1.133283 | 1.010634 | 1.270815 | 0.032276 |
| THBD | 1.148177 | 1.024355 | 1.286967 | 0.017631 |
| GIMAP5 | 1.049803 | 0.942392 | 1.169457 | 0.377479 |
| FCER1G | 1.097461 | 0.97776 | 1.231816 | 0.114503 |
| CLEC4E | 1.100785 | 0.986105 | 1.228802 | 0.087138 |
| CDGAP | 1.065964 | 0.946311 | 1.200746 | 0.293007 |
| ABI3 | 1.101502 | 0.980093 | 1.237949 | 0.104697 |
| ARHGAP9 | 1.01923 | 0.909423 | 1.142297 | 0.743288 |
| TRIM22 | 1.067299 | 0.954807 | 1.193043 | 0.251733 |
| ABCA6 | 1.150026 | 1.024867 | 1.290468 | 0.017416 |
| DOCK2 | 1.039061 | 0.926454 | 1.165355 | 0.512656 |
| FMO1 | 1.208086 | 1.083833 | 1.346583 | 0.000641 |
| IL16 | 1.025604 | 0.913592 | 1.151349 | 0.668331 |
| PTGER2 | 1.189257 | 1.058504 | 1.336162 | 0.003537 |
| PSCDBP | 0.997639 | 0.893227 | 1.114256 | 0.966572 |
| GPR18 | 1.082421 | 0.969353 | 1.208677 | 0.159428 |
| IRF8 | 1.052748 | 0.943554 | 1.174577 | 0.35755 |
| CENTA2 | 1.112853 | 0.992982 | 1.247195 | 0.065938 |
| CCL2 | 1.059047 | 0.948507 | 1.182469 | 0.307721 |
| TDO2 | 1.053354 | 0.940759 | 1.179424 | 0.36749 |
| CDH11 | 1.127536 | 1.0006 | 1.270575 | 0.04886 |
| FRMD6 | 1.164877 | 1.041571 | 1.30278 | 0.007507 |
| SPATA9 | 1.064117 | 0.947738 | 1.194787 | 0.292971 |
| LHFPL2 | 1.17535 | 1.042739 | 1.324827 | 0.008166 |
| PLXDC1 | 1.108085 | 0.995515 | 1.233383 | 0.060419 |
| SRPX2 | 1.131127 | 1.006477 | 1.271215 | 0.038608 |
| PDLIM3 | 1.130465 | 1.010551 | 1.264608 | 0.03208 |
| RGS1 | 1.116851 | 0.998 | 1.249855 | 0.054217 |
| CD37 | 1.042537 | 0.930723 | 1.167785 | 0.471729 |
| CXCL12 | 1.071657 | 0.949611 | 1.20939 | 0.261928 |
| COL10A1 | 1.14097 | 1.019152 | 1.277349 | 0.022063 |
| TREM1 | 1.234117 | 1.096578 | 1.388908 | 0.000484 |
| MEF2C | 1.162154 | 1.032433 | 1.308174 | 0.012828 |
| IL2RA | 1.071764 | 0.953107 | 1.205193 | 0.246987 |
| TSHZ3 | 1.156048 | 1.032889 | 1.293891 | 0.011636 |
| KCND2 | 1.198699 | 1.07772 | 1.333257 | 0.000841 |
| PDPN | 1.178083 | 1.052801 | 1.318273 | 0.004278 |
| CD180 | 1.032088 | 0.9209 | 1.1567 | 0.587083 |
| MPEG1 | 1.118782 | 1.001718 | 1.249527 | 0.046546 |
| FTHL17 | 1.140916 | 1.015137 | 1.282279 | 0.026963 |
| FCGR3A | 1.118409 | 0.997207 | 1.254344 | 0.055855 |
| SIGLEC7 | 1.104177 | 0.985075 | 1.237679 | 0.088803 |
| POSTN | 1.099075 | 0.97623 | 1.237379 | 0.11825 |
| COL6A3 | 1.066127 | 0.950106 | 1.196316 | 0.276025 |
| URP2 | 1.007211 | 0.897309 | 1.130574 | 0.902988 |
| CCL11 | 1.150976 | 1.025549 | 1.291744 | 0.016917 |
| ADORA3 | 1.287226 | 1.144277 | 1.448033 | 2.62E-05 |
| C17orf60 | 1.079084 | 0.963561 | 1.208457 | 0.187686 |
| ACSL1 | 1.117743 | 1.001894 | 1.246988 | 0.046168 |
| CD93 | 1.171816 | 1.043844 | 1.315477 | 0.007205 |
| IL18RAP | 1.097916 | 0.978039 | 1.232486 | 0.113301 |
| KIAA1949 | 1.053955 | 0.938026 | 1.184211 | 0.376764 |
| PGDS | 1.259158 | 1.124557 | 1.40987 | 6.47E-05 |
| FCGR1A | 1.103936 | 0.986578 | 1.235254 | 0.084648 |
| COPZ2 | 1.112914 | 0.991973 | 1.2486 | 0.068354 |
| SLC31A2 | 1.114409 | 0.989912 | 1.254564 | 0.073099 |
| TGFBR2 | 1.212419 | 1.078828 | 1.362552 | 0.001221 |
| COLEC12 | 1.152586 | 1.030311 | 1.289373 | 0.013071 |
| CTSS | 1.047782 | 0.933548 | 1.175994 | 0.42808 |
| BCL2A1 | 1.025919 | 0.910281 | 1.156247 | 0.674945 |
| CD68 | 1.090975 | 0.972409 | 1.223998 | 0.137986 |
| CTHRC1 | 1.207017 | 1.068595 | 1.36337 | 0.002466 |
| LACTB | 1.150425 | 1.024079 | 1.292359 | 0.018235 |
| TBXAS1 | 1.141117 | 1.015875 | 1.281799 | 0.026048 |
| MMP19 | 1.128699 | 1.010386 | 1.260866 | 0.032125 |
| MARCO | 1.120893 | 1.000534 | 1.25573 | 0.048934 |
| FNDC1 | 1.133382 | 1.011563 | 1.269871 | 0.030917 |
| SPRED1 | 1.128169 | 1.007102 | 1.263789 | 0.037328 |
| OMD | 1.169967 | 1.048382 | 1.305653 | 0.005049 |
| CFHR3 | 1.082562 | 0.965893 | 1.213322 | 0.17272 |
| FAM26E | 1.121228 | 0.996732 | 1.261274 | 0.056722 |
| WIPF1 | 1.029985 | 0.91769 | 1.156021 | 0.61594 |
| COL1A2 | 1.134125 | 1.006237 | 1.278267 | 0.039224 |
| GLS2 | 0.878107 | 0.781228 | 0.986999 | 0.029305 |
| GIMAP2 | 1.123677 | 1.006727 | 1.254213 | 0.037569 |
| SH2B3 | 1.122871 | 1.004282 | 1.255463 | 0.041852 |
| SLC7A7 | 1.111755 | 0.991201 | 1.24697 | 0.070443 |
| SFRP4 | 1.110455 | 0.986755 | 1.249661 | 0.082088 |
| RCSD1 | 1.00632 | 0.901752 | 1.123014 | 0.91039 |
| OLFML1 | 1.201658 | 1.072691 | 1.34613 | 0.001517 |
| FGR | 1.097614 | 0.985795 | 1.222117 | 0.089321 |
| ETV1 | 1.09915 | 0.979786 | 1.233056 | 0.107005 |
| ITGA11 | 1.099633 | 0.983572 | 1.229388 | 0.095138 |
| IGSF21 | 1.228765 | 1.089688 | 1.385594 | 0.000775 |
| CASP1 | 1.084289 | 0.970316 | 1.21165 | 0.153243 |
| ASPN | 1.161651 | 1.035171 | 1.303585 | 0.010844 |
| FZD1 | 1.246284 | 1.102827 | 1.408401 | 0.000418 |
| OSM | 1.164276 | 1.041132 | 1.301986 | 0.007661 |
| P2RY14 | 1.011233 | 0.906325 | 1.128284 | 0.841565 |
| ATP6V1B2 | 1.202927 | 1.071807 | 1.350088 | 0.001703 |
| CH25H | 1.225144 | 1.088721 | 1.37866 | 0.000748 |
| CD300A | 1.111203 | 0.987971 | 1.249806 | 0.078718 |
| CCR2 | 0.990592 | 0.884552 | 1.109343 | 0.87002 |
| ANTXR1 | 1.150299 | 1.02114 | 1.295795 | 0.02121 |
| HNT | 1.11145 | 0.989468 | 1.24847 | 0.074837 |
| EDNRA | 1.130939 | 1.010265 | 1.266028 | 0.032568 |
| PTGER3 | 1.196833 | 1.07053 | 1.338038 | 0.00159 |
| CASP5 | 1.065509 | 0.954096 | 1.189933 | 0.260143 |
| P2RY5 | 1.150595 | 1.031881 | 1.282967 | 0.011576 |
| CATSPER1 | 1.150146 | 1.024123 | 1.291676 | 0.01815 |
| COL8A1 | 1.133846 | 1.007635 | 1.275865 | 0.036953 |
| PODN | 1.232785 | 1.092975 | 1.390479 | 0.000656 |
| FAM78A | 1.017642 | 0.90898 | 1.139294 | 0.761474 |
| EPYC | 1.093349 | 0.978021 | 1.222276 | 0.116602 |
| SPI1 | 1.070888 | 0.955178 | 1.200616 | 0.240422 |
| EDIL3 | 1.105398 | 0.990745 | 1.233319 | 0.072886 |
| B2M | 1.129315 | 1.00908 | 1.263875 | 0.034231 |
| INHBA | 1.072241 | 0.95478 | 1.204153 | 0.238687 |
| TRPV2 | 0.999902 | 0.890737 | 1.122447 | 0.998678 |
| PIK3AP1 | 1.098126 | 0.986695 | 1.222141 | 0.086417 |
| C13orf15 | 1.256641 | 1.120854 | 1.408877 | 9.02E-05 |
| CD163 | 1.170757 | 1.04462 | 1.312124 | 0.006718 |
| ADAM12 | 1.111522 | 0.984929 | 1.254385 | 0.086562 |
| LHFP | 1.179442 | 1.045907 | 1.330026 | 0.0071 |
| SQRDL | 1.072178 | 0.957722 | 1.200312 | 0.226289 |
| ANXA2 | 1.241995 | 1.096819 | 1.406386 | 0.000633 |
| NCF1 | 1.047922 | 0.934338 | 1.175315 | 0.423896 |
| MAFB | 1.127407 | 1.002312 | 1.268115 | 0.045668 |
| C1QB | 1.143946 | 1.020679 | 1.282101 | 0.020788 |
| RASGRP3 | 1.097623 | 0.980415 | 1.228842 | 0.105948 |
| CYB5R4 | 1.050573 | 0.93453 | 1.181027 | 0.40873 |
| GREM1 | 1.119505 | 0.998047 | 1.255745 | 0.05403 |
| EHD2 | 1.205867 | 1.063809 | 1.366894 | 0.00342 |
| FTL | 1.056245 | 0.942919 | 1.18319 | 0.344674 |
| ITGAX | 0.989903 | 0.87879 | 1.115066 | 0.867328 |
| FCER1A | 1.171075 | 1.050734 | 1.305199 | 0.004311 |
| LILRA2 | 1.225271 | 1.084666 | 1.384102 | 0.001088 |
| GALNACT-2 | 1.145853 | 1.021502 | 1.285343 | 0.020183 |
| SULF1 | 1.065637 | 0.949379 | 1.196132 | 0.280764 |
| GPR1 | 1.118724 | 0.992545 | 1.260942 | 0.066149 |
| C13orf33 | 1.097676 | 0.980373 | 1.229015 | 0.106049 |
| MDFIC | 1.092142 | 0.974774 | 1.223642 | 0.128635 |
| AP1S2 | 1.03601 | 0.921915 | 1.164225 | 0.55234 |
| IKZF1 | 0.958635 | 0.857884 | 1.071217 | 0.45587 |
| KCNMB1 | 1.120071 | 0.996142 | 1.259417 | 0.058045 |
| SIRPA | 1.150588 | 1.022372 | 1.294884 | 0.019965 |
| ARHGAP30 | 1.006193 | 0.897289 | 1.128313 | 0.915876 |
| LILRA1 | 1.163275 | 1.037693 | 1.304055 | 0.009466 |
| GLT8D2 | 1.186521 | 1.055389 | 1.333946 | 0.004208 |
| DRAM | 1.096773 | 0.978887 | 1.228855 | 0.111351 |
| GIMAP7 | 1.030274 | 0.922971 | 1.150051 | 0.595076 |
| IL10 | 1.16213 | 1.042586 | 1.29538 | 0.006668 |
| CCL3 | 1.132032 | 1.011692 | 1.266686 | 0.030566 |
| HTRA1 | 1.17649 | 1.040686 | 1.330017 | 0.009398 |
| LILRB2 | 1.040777 | 0.93452 | 1.159116 | 0.46697 |
| HLA-DPB1 | 1.01171 | 0.911463 | 1.122984 | 0.826898 |
| HTRA4 | 1.007741 | 0.890324 | 1.140643 | 0.902904 |
| LOXL2 | 1.137682 | 1.011826 | 1.279193 | 0.031044 |
| CYLD | 1.050069 | 0.932722 | 1.18218 | 0.419066 |
| ACTA2 | 1.060685 | 0.942665 | 1.193481 | 0.327627 |
| BIN2 | 0.998895 | 0.889991 | 1.121125 | 0.985029 |
| C13orf18 | 1.063132 | 0.947752 | 1.192557 | 0.29628 |
| ITGBL1 | 1.09759 | 0.978447 | 1.231241 | 0.112218 |
| C3orf64 | 1.086316 | 0.968035 | 1.219051 | 0.159243 |
| MYO1F | 1.033694 | 0.92005 | 1.161374 | 0.577062 |
| PIK3CG | 1.130827 | 1.007579 | 1.26915 | 0.036779 |
| LST1 | 1.036599 | 0.929873 | 1.155575 | 0.516716 |
| ITGB6 | 1.135384 | 1.00905 | 1.277535 | 0.034888 |
| SLC12A8 | 1.164388 | 1.035391 | 1.309457 | 0.011069 |
| CLIC2 | 1.097544 | 0.976397 | 1.233723 | 0.118832 |
| GZMK | 1.022375 | 0.916102 | 1.140977 | 0.692724 |
| ANKRD22 | 1.012389 | 0.9022 | 1.136037 | 0.83411 |
| NEDD4 | 1.09881 | 0.976785 | 1.236079 | 0.116677 |
| PLS3 | 1.032184 | 0.928921 | 1.146927 | 0.555859 |
| HNMT | 1.141767 | 1.014958 | 1.28442 | 0.027304 |
| PHLDB2 | 1.17095 | 1.046522 | 1.310171 | 0.0059 |
| SLC2A5 | 1.111883 | 0.987084 | 1.252461 | 0.08082 |
| RUNX2 | 1.087311 | 0.972606 | 1.215544 | 0.141119 |
| HMOX1 | 1.057236 | 0.943012 | 1.185295 | 0.340026 |
| HOPX | 1.142274 | 1.026164 | 1.271521 | 0.015007 |
| SLC46A3 | 1.092026 | 0.97482 | 1.223324 | 0.128581 |
| LDB2 | 1.100028 | 0.981581 | 1.232767 | 0.100978 |
| HEPH | 1.085252 | 0.965926 | 1.21932 | 0.16863 |
| OSCAR | 1.025656 | 0.915458 | 1.149119 | 0.662242 |
| LOX | 1.123472 | 0.996946 | 1.266056 | 0.056161 |
| IFNGR1 | 1.17541 | 1.052313 | 1.312905 | 0.004192 |
| RARRES1 | 1.174779 | 1.048728 | 1.31598 | 0.00541 |
| FBN1 | 1.087308 | 0.962376 | 1.228458 | 0.178904 |
| DOK3 | 1.063884 | 0.946976 | 1.195225 | 0.297109 |
| PDCD1LG2 | 1.052688 | 0.933831 | 1.186673 | 0.400909 |
| SIGLEC5 | 1.163572 | 1.037616 | 1.304818 | 0.009551 |
| TRAF3IP3 | 0.958136 | 0.861059 | 1.066157 | 0.43267 |
| STAB1 | 1.200223 | 1.070737 | 1.345367 | 0.001728 |
| IL2RB | 1.011773 | 0.903068 | 1.133564 | 0.840052 |
| FAM113B | 0.968848 | 0.86437 | 1.085955 | 0.586719 |
| FASLG | 1.073342 | 0.957045 | 1.20377 | 0.226428 |
| CCR1 | 1.007753 | 0.903273 | 1.124317 | 0.890008 |
| SELP | 1.170293 | 1.04119 | 1.315403 | 0.00837 |
| CALCRL | 1.109079 | 0.999674 | 1.230458 | 0.050723 |
| PPEF1 | 1.09239 | 0.973415 | 1.225907 | 0.133102 |
| SPON2 | 1.121327 | 1.00148 | 1.255515 | 0.047077 |
| SCG2 | 1.129239 | 1.010793 | 1.261565 | 0.031568 |
| GBP2 | 0.964266 | 0.867557 | 1.071755 | 0.49979 |
| C16orf30 | 1.120871 | 0.998039 | 1.258821 | 0.054003 |
| AGTR1 | 1.128222 | 1.01646 | 1.252272 | 0.023408 |
| CXorf36 | 1.186289 | 1.062535 | 1.324456 | 0.002373 |
| SLAMF1 | 0.969678 | 0.864064 | 1.088202 | 0.600747 |
| PDGFRA | 1.202892 | 1.060602 | 1.364271 | 0.004028 |
| GPRIN3 | 1.03409 | 0.917031 | 1.166093 | 0.584451 |
| P2RY12 | 1.240384 | 1.113635 | 1.381558 | 8.97E-05 |
| HEG1 | 1.080591 | 0.961039 | 1.215015 | 0.195095 |
| CCDC80 | 1.181415 | 1.04732 | 1.332678 | 0.006685 |
| TNFSF4 | 1.14656 | 1.017451 | 1.292053 | 0.024846 |
| COL1A1 | 1.071283 | 0.953163 | 1.204042 | 0.248011 |
| CD2 | 0.977006 | 0.872321 | 1.094253 | 0.687469 |
| TMEM71 | 1.065012 | 0.94903 | 1.195168 | 0.284311 |
| SEC24D | 1.105076 | 0.991051 | 1.23222 | 0.072149 |
| ZNF469 | 1.137239 | 1.016416 | 1.272425 | 0.024827 |
| PALLD | 1.196683 | 1.065008 | 1.344637 | 0.002537 |
| FGF1 | 1.114636 | 0.992342 | 1.252003 | 0.067204 |
| LRRC8C | 1.067477 | 0.956055 | 1.191884 | 0.245659 |
| ECGF1 | 1.045107 | 0.932158 | 1.171741 | 0.449614 |
| NBL1 | 1.214309 | 1.076402 | 1.369883 | 0.001594 |
| MSN | 1.035923 | 0.924856 | 1.160327 | 0.541908 |
| LGALS1 | 1.125799 | 0.996618 | 1.271724 | 0.056717 |
| STK17B | 1.124049 | 0.999968 | 1.263527 | 0.050062 |
| MAN2A1 | 1.120665 | 1.002066 | 1.253301 | 0.045921 |
| PAG1 | 1.039018 | 0.926144 | 1.165649 | 0.514187 |
| AEBP1 | 1.16756 | 1.035393 | 1.316598 | 0.011491 |
| ADAM8 | 1.087626 | 0.967444 | 1.222738 | 0.159736 |
| HS3ST1 | 1.105208 | 0.990829 | 1.23279 | 0.072706 |
| SELPLG | 1.064536 | 0.946758 | 1.196965 | 0.295835 |
| ECM1 | 1.073497 | 0.955669 | 1.205852 | 0.231863 |
| RAB27A | 1.014562 | 0.907518 | 1.134233 | 0.799393 |
| CCL23 | 1.094981 | 0.973166 | 1.232045 | 0.131573 |
| GGTLA1 | 1.12096 | 0.995885 | 1.261743 | 0.058537 |
| CAV2 | 1.148978 | 1.02015 | 1.294075 | 0.022093 |
| AAAS | 0.826279 | 0.731899 | 0.932831 | 0.002046 |
| MYO5A | 1.153258 | 1.026759 | 1.295341 | 0.016153 |
| TMEM176B | 1.080837 | 0.970274 | 1.204 | 0.157987 |
| P4HA3 | 1.100793 | 0.979049 | 1.237676 | 0.108298 |
| INMT | 1.230925 | 1.097759 | 1.380246 | 0.000376 |
| PTAFR | 1.148023 | 1.021692 | 1.289975 | 0.0203 |
| NID2 | 1.120868 | 0.998008 | 1.258852 | 0.054067 |
| ASAHL | 1.183988 | 1.049935 | 1.335155 | 0.005873 |
| CD28 | 1.038153 | 0.926009 | 1.163878 | 0.520888 |
| NEXN | 1.134041 | 1.006614 | 1.277599 | 0.038607 |
| ACTR3 | 1.090844 | 0.978783 | 1.215735 | 0.115902 |
| DOCK8 | 0.993301 | 0.883864 | 1.116289 | 0.910147 |
| CCL4 | 1.12405 | 1.006526 | 1.255296 | 0.037948 |
| ESAM | 1.07242 | 0.961474 | 1.196168 | 0.209534 |
| ELTD1 | 1.151008 | 1.031231 | 1.284696 | 0.012124 |
| C4orf32 | 1.056259 | 0.93863 | 1.18863 | 0.363562 |
| KIAA0247 | 1.096778 | 0.972497 | 1.23694 | 0.132203 |
| ADAMTSL3 | 1.061572 | 0.942947 | 1.19512 | 0.323007 |
| C13orf31 | 1.190834 | 1.057125 | 1.341455 | 0.004051 |
| C5AR1 | 1.276669 | 1.143627 | 1.42519 | 1.36E-05 |
| STX12 | 1.21306 | 1.085294 | 1.355868 | 0.00067 |
| PTGIR | 1.096374 | 0.972539 | 1.235978 | 0.132423 |
| C17orf87 | 1.153405 | 1.030878 | 1.290495 | 0.01275 |
| ARNTL | 1.112816 | 0.992739 | 1.247416 | 0.066524 |
| SELE | 1.112735 | 0.988617 | 1.252436 | 0.076688 |
| PLA2G4C | 1.117293 | 0.995212 | 1.254349 | 0.060291 |
| PDE4B | 0.977873 | 0.870465 | 1.098535 | 0.706236 |
| 8-Sep | 1.142477 | 1.008889 | 1.293753 | 0.035777 |
| ZCCHC5 | 1.191815 | 1.062007 | 1.337489 | 0.002859 |
| IL7 | 1.060316 | 0.949274 | 1.184347 | 0.299436 |
| VNN2 | 1.119032 | 0.999482 | 1.252881 | 0.05106 |
| LRRK2 | 1.017933 | 0.909512 | 1.139278 | 0.757074 |
| JAK2 | 0.998825 | 0.886251 | 1.125698 | 0.984623 |
| LAMA4 | 1.038455 | 0.929953 | 1.159617 | 0.502747 |
| SVEP1 | 1.081181 | 0.963095 | 1.213746 | 0.185927 |
| CD80 | 1.065369 | 0.954721 | 1.188841 | 0.257728 |
| EFEMP1 | 1.128622 | 1.001097 | 1.272391 | 0.047942 |
| MAN1A1 | 1.04376 | 0.934013 | 1.166403 | 0.449876 |
| MYL6B | 0.860393 | 0.759319 | 0.97492 | 0.018357 |
| MST150 | 1.183426 | 1.051685 | 1.33167 | 0.00516 |
| TPST2 | 1.050051 | 0.938827 | 1.174452 | 0.39258 |
| 4-Sep | 1.136578 | 1.011689 | 1.276884 | 0.031111 |
| RAC2 | 0.998526 | 0.896307 | 1.112403 | 0.978645 |
| FBXO32 | 1.103595 | 0.986464 | 1.234634 | 0.085088 |
| ACVRL1 | 1.143588 | 1.018478 | 1.284068 | 0.023226 |
| HLA-DPA1 | 1.041629 | 0.936611 | 1.158422 | 0.451933 |
| CCRL1 | 1.107629 | 0.995759 | 1.232067 | 0.059873 |
| DPT | 1.177971 | 1.045951 | 1.326656 | 0.006919 |
| DPP4 | 1.106094 | 0.984213 | 1.243067 | 0.090489 |
| P2RY6 | 1.051481 | 0.938874 | 1.177593 | 0.385065 |
| CD86 | 1.210855 | 1.076224 | 1.362328 | 0.001465 |
| COL5A1 | 1.120007 | 0.996965 | 1.258233 | 0.056291 |
| MGAT4A | 1.090287 | 0.970643 | 1.224679 | 0.144966 |
| TREM2 | 1.189105 | 1.053844 | 1.341727 | 0.004936 |
| MLKL | 1.019139 | 0.910657 | 1.140545 | 0.741287 |
| MRVI1 | 1.17661 | 1.048814 | 1.319978 | 0.005565 |
| SEC23A | 1.137647 | 1.007484 | 1.284627 | 0.037503 |
| SPHK1 | 1.075939 | 0.959354 | 1.206692 | 0.210994 |
| RFTN1 | 1.077707 | 0.957271 | 1.213295 | 0.215818 |
| SIGLEC10 | 1.133549 | 1.016575 | 1.263983 | 0.024084 |
| CBLN4 | 1.167392 | 1.031613 | 1.321042 | 0.014155 |
| CRISPLD2 | 1.101662 | 0.98082 | 1.237393 | 0.102413 |
| HS3ST3B1 | 1.146703 | 1.022015 | 1.286603 | 0.019768 |
| TBX21 | 1.042286 | 0.931296 | 1.166502 | 0.470944 |
| RHOH | 0.951528 | 0.850776 | 1.064211 | 0.384233 |
| MYLK | 1.058597 | 0.941611 | 1.190118 | 0.340567 |
| OSTM1 | 1.102653 | 0.981966 | 1.238173 | 0.098482 |
| GPR171 | 0.986769 | 0.878789 | 1.108017 | 0.821779 |
| AQP1 | 1.114517 | 0.988629 | 1.256435 | 0.076236 |
| CCDC102B | 1.083885 | 0.97167 | 1.209059 | 0.148577 |
| EGR2 | 1.150735 | 1.028358 | 1.287676 | 0.014389 |
| DOCK4 | 1.186631 | 1.057858 | 1.331081 | 0.003504 |
| SIGLEC1 | 1.119457 | 0.998294 | 1.255325 | 0.053514 |
| TIMD4 | 1.029278 | 0.918527 | 1.153382 | 0.61931 |
| TMEM46 | 1.054161 | 0.940074 | 1.182093 | 0.366772 |
| LIPA | 1.173683 | 1.048147 | 1.314254 | 0.005525 |
| NYD-SP21 | 1.184528 | 1.05541 | 1.329442 | 0.00403 |
| TCEAL7 | 1.155836 | 1.028968 | 1.298347 | 0.014633 |
| CNTN1 | 1.155308 | 1.034856 | 1.289781 | 0.010174 |
| COL16A1 | 1.175272 | 1.046968 | 1.319299 | 0.006179 |
| LILRA4 | 1.021267 | 0.909264 | 1.147066 | 0.722543 |
| STAT4 | 0.944981 | 0.843995 | 1.05805 | 0.326397 |
| CLEC10A | 1.027628 | 0.917161 | 1.151402 | 0.638576 |
| CD300C | 1.06386 | 0.944572 | 1.198213 | 0.307633 |
| P2RY10 | 0.960562 | 0.855014 | 1.079138 | 0.498075 |
| LIMA1 | 1.086066 | 0.967714 | 1.218893 | 0.160775 |
| MYCT1 | 1.144807 | 1.022688 | 1.281507 | 0.018785 |
| RASGRF2 | 1.065848 | 0.946347 | 1.20044 | 0.293232 |
| PRRX1 | 1.093663 | 0.977713 | 1.223364 | 0.117397 |
| TBC1D8B | 1.231741 | 1.087629 | 1.394947 | 0.001027 |
| PQLC3 | 1.121334 | 1.002616 | 1.254109 | 0.044886 |
| IL18 | 1.0953 | 0.979136 | 1.225245 | 0.111529 |
| C5orf20 | 1.003684 | 0.899515 | 1.119917 | 0.947556 |
| GPR116 | 1.119026 | 1.000045 | 1.252162 | 0.049908 |
| CD244 | 1.010596 | 0.897409 | 1.13806 | 0.861925 |
| LCP1 | 0.977421 | 0.872822 | 1.094555 | 0.692492 |
| EDG1 | 1.071017 | 0.95748 | 1.198017 | 0.230142 |
| GADD45B | 1.130727 | 1.006837 | 1.269862 | 0.037983 |
| FOLR2 | 1.18479 | 1.060194 | 1.324028 | 0.00278 |
| COL11A1 | 1.140526 | 1.0156 | 1.280818 | 0.026317 |
| COP1 | 1.020727 | 0.91286 | 1.14134 | 0.718838 |
| ADAMTS12 | 1.090901 | 0.96748 | 1.230066 | 0.155528 |
| PYHIN1 | 1.007499 | 0.904555 | 1.122158 | 0.891938 |
| NR3C1 | 1.080322 | 0.962791 | 1.2122 | 0.18861 |
| PELP1 | 0.829487 | 0.734027 | 0.937361 | 0.002727 |
| C20orf82 | 1.137183 | 1.013857 | 1.275509 | 0.028168 |
| INCA | 1.024293 | 0.915831 | 1.1456 | 0.674254 |
| CD74 | 1.10393 | 0.986039 | 1.235917 | 0.086169 |
| PSTPIP2 | 1.031353 | 0.917394 | 1.159469 | 0.605321 |
| ASAM | 1.103021 | 0.981686 | 1.239353 | 0.099127 |
| COL6A2 | 1.097184 | 0.975268 | 1.234341 | 0.122768 |
| TM6SF1 | 1.097764 | 0.977122 | 1.233301 | 0.116337 |
| GZMH | 1.042428 | 0.933076 | 1.164594 | 0.462407 |
| AK5 | 1.134843 | 1.022422 | 1.259626 | 0.017474 |
| SH3GLB1 | 1.041097 | 0.934412 | 1.159961 | 0.465306 |
| CD69 | 1.021318 | 0.914339 | 1.140813 | 0.708667 |
| SPARCL1 | 1.17309 | 1.047063 | 1.314285 | 0.005904 |
| CFD | 1.107549 | 0.981213 | 1.25015 | 0.098321 |
| SIRPG | 0.999198 | 0.893894 | 1.116907 | 0.988734 |
| PRKG1 | 1.196416 | 1.07502 | 1.33152 | 0.001019 |
| C10orf56 | 1.192516 | 1.057525 | 1.344737 | 0.004073 |
| PLN | 1.149385 | 1.023692 | 1.290512 | 0.018461 |
| DNAJC5B | 1.03224 | 0.922245 | 1.155353 | 0.580981 |
| ERG | 1.037528 | 0.921741 | 1.167859 | 0.541729 |
| PROS1 | 1.113822 | 0.995518 | 1.246185 | 0.059896 |
| S100A9 | 1.073419 | 0.958774 | 1.201773 | 0.218916 |
| GNB4 | 1.118268 | 0.998762 | 1.252074 | 0.052565 |
| IL4R | 1.061831 | 0.944331 | 1.193951 | 0.316013 |
| HLA-DRA | 1.078637 | 0.968298 | 1.20155 | 0.169177 |
| OSMR | 1.083252 | 0.967304 | 1.213099 | 0.166221 |
| Rgr | 0.955255 | 0.849002 | 1.074807 | 0.446734 |
| FAM20A | 0.995393 | 0.886643 | 1.117482 | 0.93765 |
| EMCN | 1.141553 | 1.022403 | 1.274589 | 0.018578 |
| CHERP | 0.924274 | 0.816418 | 1.046379 | 0.213551 |
| RAP1A | 1.061872 | 0.944523 | 1.1938 | 0.315026 |
| MTMR6 | 1.228034 | 1.100252 | 1.370657 | 0.000248 |
| CCL5 | 0.999652 | 0.893203 | 1.118788 | 0.995168 |
| ADAMDEC1 | 1.036489 | 0.92514 | 1.161239 | 0.536528 |
| PGCP | 1.162711 | 1.041721 | 1.297754 | 0.007166 |
| ACSL4 | 1.060123 | 0.945663 | 1.188436 | 0.316556 |
| CMTM3 | 1.115505 | 0.991344 | 1.255217 | 0.069437 |
| ASAH1 | 1.312324 | 1.169474 | 1.472622 | 3.79E-06 |
| DACT1 | 1.11664 | 0.995237 | 1.252852 | 0.06029 |
| PRKCH | 1.047027 | 0.936706 | 1.170341 | 0.418539 |
| YIPF5 | 1.147662 | 1.023074 | 1.287421 | 0.018822 |
| CD247 | 0.93886 | 0.835586 | 1.054898 | 0.288648 |
| C12orf10 | 0.918098 | 0.816286 | 1.032608 | 0.154186 |
| RASSF2 | 1.106367 | 0.983208 | 1.244953 | 0.093207 |
| SLC16A3 | 1.144033 | 1.019842 | 1.283346 | 0.021728 |
| SH3BGRL | 1.09361 | 0.981933 | 1.217988 | 0.10348 |
| GBP5 | 0.991619 | 0.889784 | 1.105109 | 0.879006 |
| HERC3 | 1.240676 | 1.103798 | 1.394527 | 0.000299 |
| CCL26 | 1.067034 | 0.947965 | 1.20106 | 0.282476 |
| CD34 | 1.080687 | 0.965845 | 1.209184 | 0.175832 |
| P2RY13 | 1.156099 | 1.040678 | 1.284321 | 0.006872 |
| STOM | 1.189087 | 1.05569 | 1.33934 | 0.004336 |
| OLFML3 | 1.143094 | 1.018502 | 1.282928 | 0.023128 |
| CCL8 | 1.059628 | 0.948453 | 1.183835 | 0.305766 |
| C1QC | 1.102202 | 0.988264 | 1.229276 | 0.08048 |
| KLF2 | 1.136687 | 1.008869 | 1.280699 | 0.035287 |
| GPR132 | 1.041028 | 0.923579 | 1.173412 | 0.510322 |
| TPM1 | 1.021596 | 0.914704 | 1.14098 | 0.704758 |
| RHOA | 1.091201 | 0.980148 | 1.214837 | 0.110981 |
| AXL | 1.22097 | 1.090639 | 1.366875 | 0.000527 |
| GEM | 1.124283 | 1.001815 | 1.261722 | 0.046504 |
| PDGFRB | 1.097914 | 0.9769 | 1.233919 | 0.116944 |
| FPRL1 | 1.210848 | 1.081277 | 1.355945 | 0.000922 |
| KLF10 | 1.184546 | 1.053912 | 1.331371 | 0.004501 |
| ADAM10 | 1.11322 | 0.985703 | 1.257234 | 0.083994 |
| LILRB4 | 1.092327 | 0.978165 | 1.219813 | 0.116886 |
| FTH1 | 1.186914 | 1.052734 | 1.338197 | 0.005117 |
| PSAP | 1.10581 | 0.992823 | 1.231656 | 0.067402 |
| TPP1 | 1.084942 | 0.970876 | 1.21241 | 0.150303 |
| NLRC3 | 0.92513 | 0.824056 | 1.038603 | 0.187397 |
| KIAA1576 | 1.15633 | 1.028243 | 1.300372 | 0.015311 |
| ABCA1 | 1.20158 | 1.072006 | 1.346816 | 0.001609 |
| HLA-DQA2 | 1.088415 | 0.976734 | 1.212866 | 0.12508 |
| LOC728215 | 1.04922 | 0.941773 | 1.168925 | 0.383408 |
| EPAS1 | 1.022299 | 0.912225 | 1.145656 | 0.704369 |
| GUCY1A3 | 1.122603 | 1.004348 | 1.254781 | 0.041714 |
| ADAM19 | 0.998291 | 0.890399 | 1.119258 | 0.976623 |
| C10orf54 | 1.159483 | 1.034402 | 1.299689 | 0.011063 |
| MSRB3 | 1.090871 | 0.973665 | 1.222185 | 0.133672 |
| SOD2 | 1.057923 | 0.944919 | 1.184441 | 0.328591 |
| C6orf65 | 1.058596 | 0.942139 | 1.189448 | 0.338256 |
| DKK2 | 1.222793 | 1.093827 | 1.366965 | 0.000405 |
| HK3 | 1.061502 | 0.949166 | 1.187133 | 0.295653 |
| COL6A6 | 1.141742 | 1.021674 | 1.275919 | 0.019377 |
| IL15 | 1.159094 | 1.034283 | 1.298967 | 0.01109 |
| TNFRSF9 | 0.924778 | 0.825882 | 1.035517 | 0.175366 |
| LYZ | 1.020835 | 0.908075 | 1.147596 | 0.729879 |
| PTGIS | 1.130634 | 1.005485 | 1.271362 | 0.040233 |
| FLJ21438 | 0.95909 | 0.859308 | 1.070458 | 0.456136 |
| ADRA2A | 1.135071 | 1.011981 | 1.273132 | 0.030516 |
| KCNK13 | 1.202258 | 1.075508 | 1.343947 | 0.001193 |
| PCDH12 | 1.108141 | 0.987067 | 1.244065 | 0.081956 |
| SORCS2 | 1.166354 | 1.036802 | 1.312093 | 0.01042 |
| IGFBP7 | 1.001949 | 0.897085 | 1.119071 | 0.972467 |
| ICOS | 0.943864 | 0.841276 | 1.058962 | 0.325063 |
| RHOJ | 1.011981 | 0.894666 | 1.14468 | 0.849737 |
| IRAK3 | 1.075865 | 0.959565 | 1.206262 | 0.210274 |
| RANBP3L | 1.116996 | 0.998741 | 1.249252 | 0.052635 |
| TMEM176A | 1.06915 | 0.961114 | 1.18933 | 0.218615 |
| CCPG1 | 1.147713 | 1.029807 | 1.279118 | 0.012737 |
| BGN | 1.150876 | 1.023861 | 1.293649 | 0.018514 |
| ICAM1 | 1.064007 | 0.95066 | 1.190868 | 0.280347 |
| DDIT4L | 1.135859 | 1.014448 | 1.271801 | 0.027197 |
| ELL2 | 0.988552 | 0.882706 | 1.10709 | 0.842049 |
| MMP13 | 1.094812 | 0.977851 | 1.225764 | 0.116088 |
| KDR | 1.155137 | 1.037267 | 1.2864 | 0.008633 |
| CASC4 | 1.237808 | 1.108759 | 1.381878 | 0.000146 |
| TRAM1 | 1.17426 | 1.046485 | 1.317637 | 0.006276 |
| ALPK2 | 1.010372 | 0.903148 | 1.130326 | 0.856936 |
| PLEKHQ1 | 1.076216 | 0.959234 | 1.207464 | 0.210913 |
| CMAH | 1.061921 | 0.944125 | 1.194414 | 0.31658 |
| CYFIP1 | 1.014266 | 0.902238 | 1.140203 | 0.812501 |
| ADAM9 | 1.266426 | 1.130159 | 1.419122 | 4.77E-05 |
| CMKLR1 | 1.091051 | 0.974527 | 1.221508 | 0.130484 |
| SAT1 | 0.990521 | 0.885404 | 1.108117 | 0.867841 |
| SYTL2 | 1.092756 | 0.977245 | 1.221921 | 0.119671 |
| CCR7 | 0.903017 | 0.81119 | 1.005238 | 0.062255 |
| FMO2 | 1.271989 | 1.134907 | 1.42563 | 3.55E-05 |
| MRO | 1.105378 | 0.986019 | 1.239184 | 0.085711 |
| TTC9 | 1.099105 | 0.972591 | 1.242077 | 0.12989 |
| SEMA3C | 1.068271 | 0.944007 | 1.208892 | 0.295237 |
| PKD2L1 | 1.119108 | 0.998855 | 1.253839 | 0.052355 |
| AIM2 | 0.981727 | 0.879184 | 1.096232 | 0.743188 |
| CD72 | 1.04558 | 0.932453 | 1.172432 | 0.445517 |
| CALD1 | 1.083095 | 0.965569 | 1.214926 | 0.173172 |
| EPSTI1 | 1.129797 | 1.010415 | 1.263285 | 0.03221 |
| MCM7 | 0.870746 | 0.778753 | 0.973605 | 0.015119 |
| GPR124 | 1.087332 | 0.964862 | 1.225348 | 0.169668 |
| RUNX1 | 1.110102 | 0.987091 | 1.248443 | 0.081311 |
| DARC | 1.1649 | 1.044196 | 1.299558 | 0.006241 |
| VIM | 1.099596 | 0.976009 | 1.238833 | 0.118579 |
| FLVCR2 | 1.031025 | 0.917044 | 1.159172 | 0.609242 |
| HEXB | 1.152726 | 1.02544 | 1.295811 | 0.017276 |
| MCRS1 | 0.887193 | 0.789795 | 0.996602 | 0.04366 |
| GNGT2 | 1.094483 | 0.973302 | 1.230752 | 0.131562 |
| IL6 | 1.059383 | 0.944992 | 1.187621 | 0.322425 |
| ENOX1 | 1.170143 | 1.038706 | 1.318212 | 0.009748 |
| HERPUD1 | 1.01693 | 0.907367 | 1.139722 | 0.772855 |
| PARVA | 1.114234 | 0.989848 | 1.254251 | 0.073293 |
| GJA1 | 0.983737 | 0.87862 | 1.101429 | 0.776112 |
| GRIN3A | 1.027855 | 0.913759 | 1.156198 | 0.647201 |
| CXCL9 | 0.982591 | 0.880161 | 1.096941 | 0.754529 |
| VSTM3 | 0.966391 | 0.865519 | 1.079019 | 0.543311 |
| PTPRE | 1.083442 | 0.965063 | 1.216342 | 0.174601 |
| GALNT5 | 1.099587 | 0.979235 | 1.23473 | 0.108457 |
| SGPP1 | 1.073518 | 0.952653 | 1.209718 | 0.2444 |
| PKIB | 1.106374 | 0.996034 | 1.228938 | 0.059317 |
| IL21R | 0.90972 | 0.808942 | 1.023052 | 0.114219 |
| B3GNT2 | 1.092473 | 0.973593 | 1.225868 | 0.132408 |
| NKG7 | 1.016354 | 0.9058 | 1.1404 | 0.782483 |
| MMP11 | 1.052575 | 0.939572 | 1.17917 | 0.376545 |
| CTSH | 1.050224 | 0.938212 | 1.175608 | 0.394442 |
| CTSO | 1.176424 | 1.056685 | 1.309733 | 0.00301 |
| FCN1 | 1.063155 | 0.951213 | 1.188271 | 0.280656 |
| NRP2 | 1.028843 | 0.918462 | 1.152489 | 0.62338 |
| ASPA | 1.011601 | 0.894555 | 1.143962 | 0.854127 |
| TNFSF12 | 1.129005 | 1.006771 | 1.266079 | 0.03795 |
| C3 | 1.043795 | 0.932995 | 1.167754 | 0.454079 |
| ITGAV | 1.153015 | 1.028506 | 1.292597 | 0.014604 |
| LILRA5 | 1.064623 | 0.941246 | 1.20417 | 0.319031 |
| MPP1 | 1.041689 | 0.927332 | 1.170148 | 0.491205 |
| USP52 | 0.921492 | 0.821602 | 1.033527 | 0.16252 |
| HCLS1 | 1.041649 | 0.93303 | 1.162912 | 0.46769 |
| COL6A1 | 1.154884 | 1.031563 | 1.292947 | 0.012443 |
| HPDL | 0.853789 | 0.765046 | 0.952826 | 0.004758 |
| FLT1 | 1.01811 | 0.909791 | 1.139325 | 0.75449 |
| CD200R1 | 1.150339 | 1.029279 | 1.285637 | 0.013564 |
| NOD2 | 1.043135 | 0.92701 | 1.173806 | 0.483107 |
| ZNF683 | 1.086928 | 0.967683 | 1.220866 | 0.159755 |
| IL18BP | 0.982219 | 0.874851 | 1.102763 | 0.761307 |
| ITGB1 | 1.189841 | 1.060617 | 1.33481 | 0.003044 |
| LRRC15 | 1.135449 | 1.012058 | 1.273885 | 0.030452 |
| TMEM87B | 1.120931 | 0.999151 | 1.257553 | 0.051716 |
| ZEB1 | 1.085061 | 0.965022 | 1.220032 | 0.172331 |
| ITK | 0.901318 | 0.799712 | 1.015833 | 0.088654 |
| COL8A2 | 1.213936 | 1.083743 | 1.359769 | 0.00081 |
| PPM1M | 1.150389 | 1.024723 | 1.291465 | 0.017608 |
| CDR2 | 1.034947 | 0.922281 | 1.161378 | 0.559125 |
| EDG6 | 0.908396 | 0.808595 | 1.020515 | 0.105669 |
| PTGER4 | 1.078149 | 0.962284 | 1.207965 | 0.194567 |
| CCL18 | 0.989812 | 0.888657 | 1.102482 | 0.85231 |
| TMEM118 | 0.924944 | 0.817671 | 1.04629 | 0.214788 |
| SMOC2 | 1.086139 | 0.966973 | 1.219991 | 0.163451 |
| HHEX | 1.025955 | 0.923913 | 1.139269 | 0.631655 |
| APOC1 | 1.025082 | 0.916212 | 1.146888 | 0.665432 |
| SNX10 | 1.028018 | 0.917878 | 1.151375 | 0.632708 |
| TMEM154 | 1.09754 | 0.981027 | 1.22789 | 0.10407 |
| CD36 | 1.130868 | 1.010238 | 1.265902 | 0.032602 |
| GZMB | 1.014404 | 0.909481 | 1.131431 | 0.797396 |
| CLEC1A | 1.13595 | 1.013425 | 1.273289 | 0.028599 |
| ATP10A | 1.068127 | 0.952977 | 1.19719 | 0.257464 |
| GGTA1 | 1.182054 | 1.051319 | 1.329046 | 0.005161 |
| RAP2B | 1.017872 | 0.912659 | 1.135214 | 0.750329 |
| TM4SF18 | 1.148949 | 1.027302 | 1.285001 | 0.015028 |
| VNN1 | 1.107628 | 0.995482 | 1.232408 | 0.060542 |
| EMP3 | 1.130369 | 1.001868 | 1.275352 | 0.04656 |
| STK3 | 1.239545 | 1.108635 | 1.385913 | 0.000163 |
| CXCL6 | 1.065103 | 0.944355 | 1.20129 | 0.304247 |
| OSTF1 | 1.133411 | 1.006205 | 1.2767 | 0.039227 |
| SFRP2 | 1.154654 | 1.02653 | 1.298771 | 0.016562 |
| NRP1 | 1.051188 | 0.93928 | 1.17643 | 0.38472 |
| CXCL1 | 1.067063 | 0.950287 | 1.198189 | 0.272349 |
| SGK | 1.096676 | 0.981676 | 1.225147 | 0.102522 |
| PFAS | 0.885867 | 0.786529 | 0.997753 | 0.045821 |
| PLB1 | 1.177426 | 1.04773 | 1.323177 | 0.006088 |
| TMEM140 | 1.018418 | 0.911042 | 1.138448 | 0.748176 |
| CXCR6 | 1.001632 | 0.892857 | 1.123659 | 0.977819 |
| GALNAC4S-6ST | 1.193609 | 1.060097 | 1.343936 | 0.003453 |
| MOSPD2 | 1.057667 | 0.950132 | 1.177372 | 0.305425 |
| CXCR3 | 0.966994 | 0.863037 | 1.083473 | 0.563009 |
| DNAJB4 | 1.125257 | 1.006311 | 1.258262 | 0.038421 |
| RCAN1 | 1.146134 | 1.023826 | 1.283053 | 0.01784 |
| ACTG2 | 1.075081 | 0.95647 | 1.208402 | 0.22483 |
| HTR2B | 1.113679 | 0.996598 | 1.244515 | 0.057455 |
| CLECL1 | 1.128746 | 1.007307 | 1.264825 | 0.03704 |
| LPXN | 0.964805 | 0.854829 | 1.08893 | 0.561749 |
| CD44 | 0.943566 | 0.833922 | 1.067625 | 0.356688 |
| IL13RA1 | 1.077583 | 0.957378 | 1.212882 | 0.215646 |
| RUNX1T1 | 1.208213 | 1.073907 | 1.359315 | 0.001656 |
| RNF13 | 1.020423 | 0.914678 | 1.138394 | 0.7172 |
| PPP3CC | 1.103911 | 0.986186 | 1.235689 | 0.085761 |
| CDH5 | 1.078154 | 0.966675 | 1.202488 | 0.176593 |
| RNASEL | 1.159315 | 1.039562 | 1.292862 | 0.007874 |
| FAM3C | 1.183893 | 1.055361 | 1.328078 | 0.003991 |
| TARS2 | 0.915522 | 0.816523 | 1.026525 | 0.130634 |
| CXCL11 | 0.99841 | 0.891671 | 1.117926 | 0.977989 |
| TIMP3 | 1.127067 | 1.00368 | 1.265623 | 0.04317 |
| MEGF10 | 1.105993 | 0.986701 | 1.239708 | 0.08362 |
| NAB2 | 0.953528 | 0.840408 | 1.081873 | 0.46016 |
| GPX1 | 1.069585 | 0.955755 | 1.196973 | 0.241307 |
| TAGLN | 1.073689 | 0.954463 | 1.207809 | 0.23645 |
| C19orf44 | 1.047425 | 0.92175 | 1.190234 | 0.477387 |
| ITGA5 | 1.095336 | 0.97257 | 1.233599 | 0.133255 |
| DPEP2 | 1.030377 | 0.918263 | 1.156179 | 0.610652 |
| RASSF4 | 1.011446 | 0.907007 | 1.12791 | 0.837831 |
| MEPCE | 0.967299 | 0.861142 | 1.086543 | 0.575103 |
| CILP | 1.197137 | 1.060406 | 1.351498 | 0.00364 |
| ACVR2B | 0.920861 | 0.823534 | 1.02969 | 0.148007 |
| IL8 | 1.046446 | 0.928258 | 1.179683 | 0.457801 |
| RCN3 | 1.129498 | 1.004207 | 1.270421 | 0.042362 |
| C22orf9 | 1.065331 | 0.943073 | 1.203439 | 0.308889 |
| SKAP2 | 1.029667 | 0.922835 | 1.148866 | 0.600903 |
| ITM2A | 1.009707 | 0.911309 | 1.118729 | 0.853499 |
| C13orf16 | 1.074991 | 0.958896 | 1.205141 | 0.214921 |
| PRF1 | 1.016616 | 0.905426 | 1.141461 | 0.780358 |
| C2 | 0.968104 | 0.869438 | 1.077968 | 0.554494 |
| LSP1 | 1.01028 | 0.899109 | 1.135198 | 0.863473 |
| TMOD2 | 1.139341 | 1.021884 | 1.270298 | 0.018777 |
| HBEGF | 1.130828 | 1.009411 | 1.266849 | 0.033871 |
| CA14 | 0.896773 | 0.792886 | 1.014271 | 0.082848 |
| IQGAP1 | 1.019634 | 0.912272 | 1.139633 | 0.731956 |
| GJE1 | 0.900188 | 0.794832 | 1.019509 | 0.097776 |
| C20orf174 | 1.013873 | 0.905487 | 1.135233 | 0.811223 |
| HLA-B | 0.983562 | 0.883045 | 1.09552 | 0.763151 |
| PHF11 | 1.123114 | 1.002972 | 1.257648 | 0.044286 |
| UBE2D1 | 1.073894 | 0.95762 | 1.204286 | 0.222721 |
| APOB48R | 1.017402 | 0.90713 | 1.141078 | 0.76819 |
| SOX12 | 0.90404 | 0.800215 | 1.021336 | 0.105065 |
| IL6ST | 1.198134 | 1.069895 | 1.341744 | 0.00175 |
| NKAPL | 1.084129 | 0.976973 | 1.203038 | 0.128201 |
| SLAMF7 | 0.924644 | 0.827961 | 1.032618 | 0.164421 |
| SDCBP | 1.158218 | 1.029231 | 1.303371 | 0.014759 |
| C11orf80 | 1.118449 | 1.000588 | 1.250193 | 0.048803 |
| DPF1 | 0.887394 | 0.790341 | 0.996365 | 0.043219 |
| DKFZP564O0823 | 0.993898 | 0.887831 | 1.112636 | 0.915341 |
| MLZE | 1.058481 | 0.93836 | 1.19398 | 0.355087 |
| BST1 | 1.035622 | 0.927293 | 1.156607 | 0.534658 |
| IL33 | 1.147077 | 1.024732 | 1.28403 | 0.017102 |
| SLC28A3 | 1.137914 | 1.018073 | 1.271863 | 0.022881 |
| CALB2 | 1.127743 | 1.00043 | 1.271258 | 0.049183 |
| FAM129A | 1.003847 | 0.896226 | 1.124391 | 0.947094 |
| GNLY | 1.030209 | 0.91691 | 1.157508 | 0.616605 |
| C15orf48 | 1.090158 | 0.971184 | 1.223707 | 0.143177 |
| MSC | 1.01648 | 0.895838 | 1.153368 | 0.799825 |
| IL8RB | 1.209117 | 1.080337 | 1.353248 | 0.00095 |
| HLA-DMA | 0.998145 | 0.897789 | 1.109718 | 0.972602 |
| PTHLH | 1.043962 | 0.926079 | 1.17685 | 0.481582 |
| MMP9 | 0.932331 | 0.826981 | 1.051102 | 0.25208 |
| IGFBP4 | 1.19468 | 1.05364 | 1.354599 | 0.005518 |
| DENND3 | 1.021226 | 0.905025 | 1.152348 | 0.733254 |
| PID1 | 1.092732 | 0.974922 | 1.22478 | 0.127607 |
| GPR137B | 1.057062 | 0.947965 | 1.178715 | 0.318048 |
| CUGBP2 | 1.084105 | 0.970501 | 1.211007 | 0.152772 |
| TARBP2 | 0.869277 | 0.767811 | 0.984152 | 0.026951 |
| LTBP2 | 1.086271 | 0.968048 | 1.218932 | 0.159253 |
| C1QA | 1.088143 | 0.974284 | 1.215308 | 0.134143 |
| RGS2 | 1.088749 | 0.973346 | 1.217835 | 0.136911 |
| CTSL1 | 1.083503 | 0.962192 | 1.22011 | 0.185572 |
| RSU1 | 1.058896 | 0.939519 | 1.19344 | 0.348397 |
| NCOA4 | 1.10949 | 0.99572 | 1.23626 | 0.0598 |
| VAV1 | 1.08026 | 0.970283 | 1.202703 | 0.158754 |
| PRKD1 | 1.038965 | 0.91982 | 1.173543 | 0.538495 |
| PLEKHC1 | 1.104114 | 0.981946 | 1.241481 | 0.097833 |
| CORIN | 1.077921 | 0.970415 | 1.197337 | 0.161593 |
| FAM49A | 1.17225 | 1.045428 | 1.314458 | 0.00652 |
| SETD7 | 1.148099 | 1.020085 | 1.292178 | 0.022042 |
| OGFRL1 | 0.976942 | 0.877 | 1.088274 | 0.671814 |
| KCNE4 | 1.041492 | 0.924435 | 1.173372 | 0.503929 |
| SERPINE1 | 1.025274 | 0.910214 | 1.154878 | 0.681094 |
| SERPINB8 | 1.052232 | 0.938884 | 1.179264 | 0.381295 |
| ZNF701 | 1.04923 | 0.937009 | 1.174891 | 0.405038 |
| METTL1 | 0.866581 | 0.776046 | 0.967678 | 0.010973 |
| CTSZ | 1.031469 | 0.916781 | 1.160504 | 0.606411 |
| SPPL2A | 1.075634 | 0.962259 | 1.202368 | 0.199495 |
| MAST1 | 1.036899 | 0.921538 | 1.166701 | 0.547089 |
| SPSB1 | 1.209052 | 1.07805 | 1.355973 | 0.001177 |
| KIF26B | 1.187674 | 1.055123 | 1.336878 | 0.004391 |
| CCL13 | 1.012065 | 0.901973 | 1.135594 | 0.838277 |
| TEX11 | 1.031705 | 0.915635 | 1.162489 | 0.60825 |
| RRM2B | 1.172131 | 1.049774 | 1.308748 | 0.00475 |
| DDX11 | 0.881775 | 0.784286 | 0.991383 | 0.035315 |
| CSF1R | 1.042988 | 0.933929 | 1.164783 | 0.455107 |
| RSPRY1 | 1.03832 | 0.929542 | 1.159828 | 0.505422 |
| BCL3 | 1.089453 | 0.972499 | 1.220473 | 0.139226 |
| TMEM158 | 1.108125 | 0.989109 | 1.241461 | 0.076552 |
| MGC14376 | 1.160081 | 1.039222 | 1.294996 | 0.008161 |
| ZMAT3 | 1.128294 | 1.007799 | 1.263195 | 0.03619 |
| CD302 | 1.145265 | 1.016752 | 1.290022 | 0.025514 |
| SMPD1 | 1.046387 | 0.933558 | 1.172852 | 0.436026 |
| KMO | 1.042056 | 0.92721 | 1.171126 | 0.489281 |
| ANGPTL2 | 1.135565 | 1.007476 | 1.279939 | 0.037348 |
| ATP6V1E1 | 1.003867 | 0.899599 | 1.120221 | 0.945005 |
| GPR92 | 1.196671 | 1.064813 | 1.344858 | 0.002576 |
| CMPK | 1.082499 | 0.959732 | 1.22097 | 0.196796 |
| GBP3 | 1.034292 | 0.927687 | 1.153148 | 0.543506 |
| ZNF366 | 1.168927 | 1.046701 | 1.305426 | 0.005606 |
| LILRB5 | 1.116081 | 0.995466 | 1.25131 | 0.059825 |
| TPBG | 1.058804 | 0.944146 | 1.187386 | 0.328507 |
| RAB9A | 1.029026 | 0.921051 | 1.149659 | 0.612927 |
| NDRG1 | 1.059643 | 0.942968 | 1.190753 | 0.330386 |
| APOL3 | 1.013874 | 0.908794 | 1.131104 | 0.805053 |
| TGM2 | 1.237822 | 1.095949 | 1.398061 | 0.000592 |
| ST3GAL1 | 1.039173 | 0.923766 | 1.168997 | 0.522337 |
| SDS | 1.145181 | 1.024506 | 1.28007 | 0.017028 |
| CARD6 | 0.981937 | 0.883153 | 1.091771 | 0.736156 |
| ZFAND6 | 1.011324 | 0.901663 | 1.134323 | 0.847511 |
| TMEM173 | 1.048193 | 0.934091 | 1.176232 | 0.423451 |
| ABCC3 | 1.125801 | 1.002218 | 1.264622 | 0.045792 |
| PARVG | 1.039382 | 0.930986 | 1.160399 | 0.491841 |
| GPR4 | 1.079664 | 0.9662 | 1.206452 | 0.176051 |
| GM2A | 1.090391 | 0.968207 | 1.227994 | 0.153544 |
| CARD8 | 1.034308 | 0.92258 | 1.159566 | 0.563024 |
| ARL6IP5 | 1.02875 | 0.90985 | 1.163188 | 0.651039 |
| C1QTNF1 | 1.046398 | 0.923198 | 1.18604 | 0.477934 |
| FEZ2 | 1.041592 | 0.926984 | 1.17037 | 0.493241 |
| PLEKHF2 | 1.201974 | 1.070208 | 1.349964 | 0.001901 |
| CNTROB | 0.90007 | 0.799172 | 1.013707 | 0.082644 |
| KLRC1 | 1.102339 | 0.980572 | 1.239227 | 0.102795 |
| CANX | 1.100823 | 0.985981 | 1.229041 | 0.087486 |
| ITGA2B | 0.900826 | 0.799536 | 1.014948 | 0.086132 |
| CLEC14A | 1.128827 | 1.010776 | 1.260666 | 0.031544 |
| RABGAP1L | 1.083251 | 0.962855 | 1.218702 | 0.183429 |
| ENTPD1 | 1.127683 | 1.005268 | 1.265006 | 0.040405 |
| JAM3 | 1.09562 | 0.973602 | 1.23293 | 0.129548 |
| FAM49B | 1.026097 | 0.917469 | 1.147588 | 0.651813 |
| TPSAB1 | 0.984477 | 0.87873 | 1.102949 | 0.787276 |
| SLC46A2 | 1.144598 | 1.027607 | 1.274908 | 0.014088 |
| HMHA1 | 1.092384 | 0.974229 | 1.22487 | 0.130296 |
| ADRB2 | 1.120016 | 0.994743 | 1.261066 | 0.061086 |
| NCAPD2 | 0.913406 | 0.81086 | 1.028921 | 0.136034 |
| IBSP | 1.094794 | 0.980652 | 1.22222 | 0.106924 |
| TGFB1I1 | 1.137598 | 1.002534 | 1.290859 | 0.045585 |
| HIC1 | 1.061848 | 0.935183 | 1.205668 | 0.354469 |
| ZNF267 | 1.065368 | 0.954362 | 1.189286 | 0.259361 |
| HLA-E | 0.981629 | 0.880178 | 1.094773 | 0.739029 |
| KDELR3 | 1.115861 | 0.992742 | 1.254251 | 0.066086 |
| TRAT1 | 0.948968 | 0.844321 | 1.066585 | 0.379592 |
| PTTG1IP | 1.199905 | 1.070144 | 1.345399 | 0.001803 |
| MEOX2 | 1.127036 | 1.008848 | 1.259071 | 0.034361 |
| TXNDC15 | 1.057334 | 0.950558 | 1.176105 | 0.304694 |
| HRH1 | 1.122057 | 1.001193 | 1.257512 | 0.047652 |
| CHST11 | 0.965216 | 0.862862 | 1.079711 | 0.535906 |
| PLSCR1 | 1.045215 | 0.93049 | 1.174085 | 0.455982 |
| CEACAM4 | 1.142559 | 1.015964 | 1.284928 | 0.026128 |
| LAPTM4B | 1.101638 | 0.984384 | 1.232858 | 0.091825 |
| TNFRSF11A | 1.060248 | 0.949962 | 1.183337 | 0.29651 |
| FSTL1 | 1.098825 | 0.974318 | 1.239243 | 0.124555 |
| REXO2 | 1.134176 | 1.012458 | 1.270526 | 0.029726 |
| COTL1 | 1.048599 | 0.930595 | 1.181566 | 0.435942 |
| FHL2 | 1.116766 | 0.99855 | 1.248977 | 0.053046 |
| PDE1A | 1.049278 | 0.939752 | 1.171569 | 0.392442 |
| TMEM9B | 1.156223 | 1.036948 | 1.289217 | 0.008973 |
| C1QTNF5 | 1.124905 | 1.002957 | 1.261682 | 0.04439 |
| KLHL4 | 1.061929 | 0.941022 | 1.198371 | 0.329908 |
| CD52 | 0.999753 | 0.891952 | 1.120582 | 0.99661 |
| CAPZA2 | 1.141876 | 1.020075 | 1.27822 | 0.021147 |
| SWAP70 | 1.093119 | 0.980697 | 1.218428 | 0.107846 |
| S100A3 | 1.090167 | 0.973058 | 1.22137 | 0.136505 |
| C21orf7 | 1.047184 | 0.932501 | 1.175971 | 0.435944 |
| HSPA6 | 1.033306 | 0.917439 | 1.163808 | 0.589244 |
| RIN2 | 1.250246 | 1.119389 | 1.3964 | 7.51E-05 |
| PLA2R1 | 1.205447 | 1.072645 | 1.35469 | 0.001704 |
| A2M | 1.013244 | 0.906534 | 1.132514 | 0.816755 |
| HAND2 | 1.069283 | 0.951324 | 1.201868 | 0.261332 |
| AQP9 | 1.159159 | 1.028706 | 1.306156 | 0.015326 |
| BATF | 0.955394 | 0.851304 | 1.072212 | 0.438159 |
| FAM63B | 1.120342 | 0.998424 | 1.257147 | 0.05322 |
| SGIP1 | 1.131279 | 1.011249 | 1.265556 | 0.031129 |
| AMPD3 | 1.049891 | 0.931836 | 1.182903 | 0.423732 |
| CTSB | 1.135448 | 1.008419 | 1.278478 | 0.035865 |
| CPNE8 | 1.110156 | 0.995485 | 1.238036 | 0.060297 |
| RNF19A | 1.155467 | 1.032054 | 1.293638 | 0.012161 |

**Supplementary Table1.** Univariate Cox regression analysis and LASSO regression algorithm were applied to excavated cancer progression associated genes successively in the TCGAarray-Agilent training dataset.
