# Supplementary figures and images for: Ferroptosis-Related Gene Signature Promotes Ovarian Cancer by Influencing Immune Infiltration and Invasion
Source: J Oncol. 2021 May 26;2021:9915312. doi: 10.1155/2021/9915312 (PMC8175133; doi:10.1155/2021/9915312)

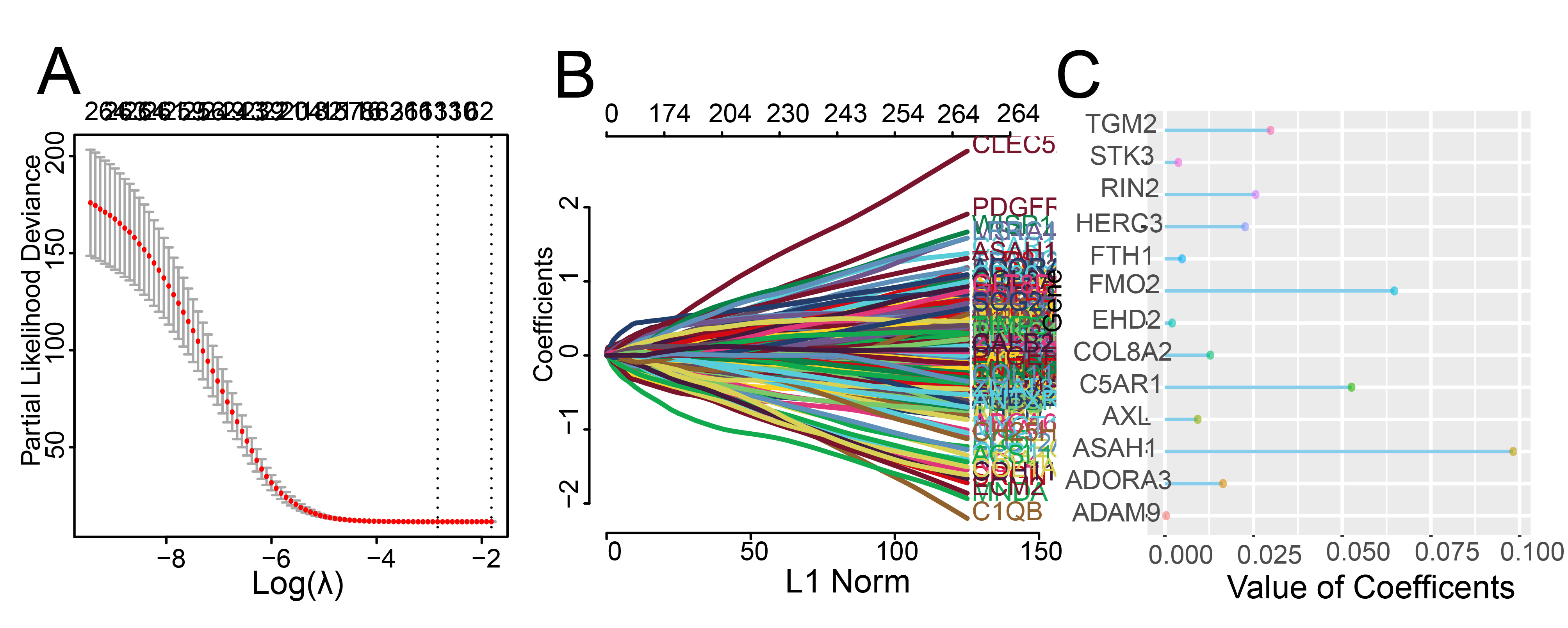

Supplement: Supplementary Materials — Supplementary Figure 1: the workflow of this article. Supplementary Figure 2: univariate Cox regression and LASSO regression were used to establish a risk score model containing 13 ferroptosis-related genes. Supplementary Figure 3: heat map and survival analysis of TCGA seq data set and ROC curve of TCGA array-Agilent data set. Supplementary Figure 4: immune infiltration analysis based on score signature in TCGA seq data set. Supplementary Figure 5: immune infiltration analysis based on score signature in TCGA array-u133a data set. Supplementary Table1: Cancer-progression-associated genes were successively excavated in the TCGA array-Agilent training data set. [file 9915312.f1.zip › 9915312.f1/supplementaty-figure 2 revised.jpg]

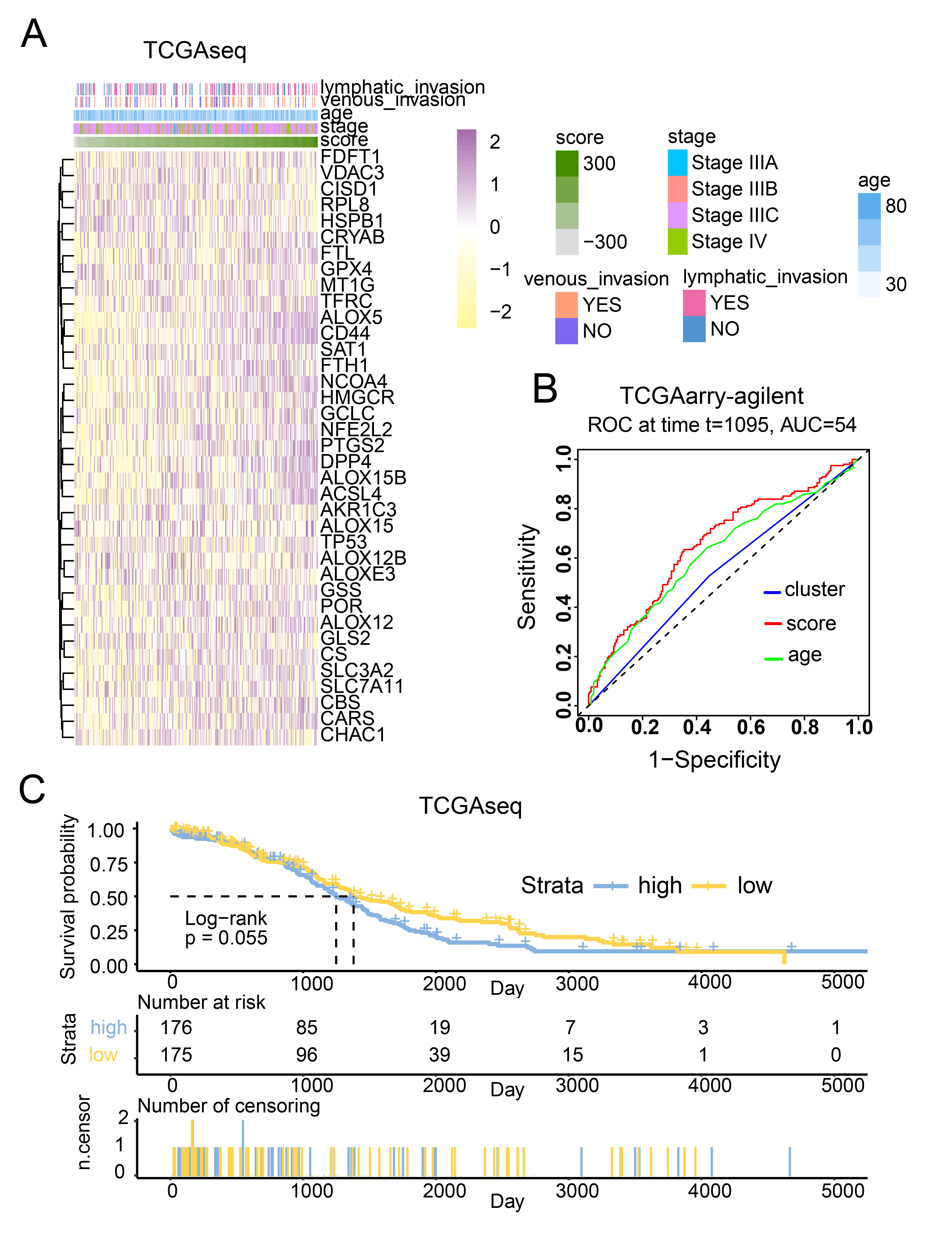

Supplement: Supplementary Materials — Supplementary Figure 1: the workflow of this article. Supplementary Figure 2: univariate Cox regression and LASSO regression were used to establish a risk score model containing 13 ferroptosis-related genes. Supplementary Figure 3: heat map and survival analysis of TCGA seq data set and ROC curve of TCGA array-Agilent data set. Supplementary Figure 4: immune infiltration analysis based on score signature in TCGA seq data set. Supplementary Figure 5: immune infiltration analysis based on score signature in TCGA array-u133a data set. Supplementary Table1: Cancer-progression-associated genes were successively excavated in the TCGA array-Agilent training data set. [file 9915312.f1.zip › 9915312.f1/supplementaty-figure 3 revised.jpg]

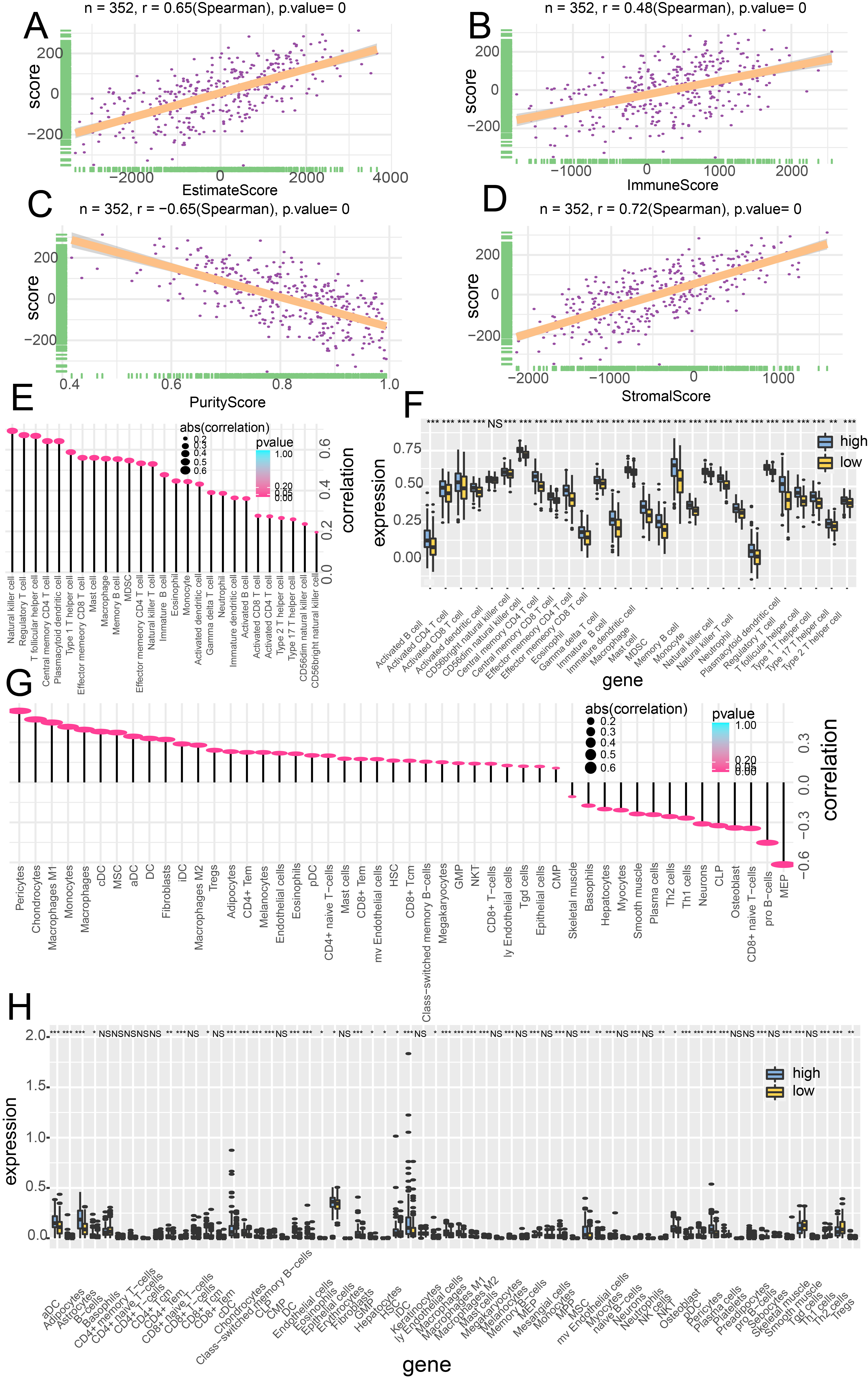

Supplement: Supplementary Materials — Supplementary Figure 1: the workflow of this article. Supplementary Figure 2: univariate Cox regression and LASSO regression were used to establish a risk score model containing 13 ferroptosis-related genes. Supplementary Figure 3: heat map and survival analysis of TCGA seq data set and ROC curve of TCGA array-Agilent data set. Supplementary Figure 4: immune infiltration analysis based on score signature in TCGA seq data set. Supplementary Figure 5: immune infiltration analysis based on score signature in TCGA array-u133a data set. Supplementary Table1: Cancer-progression-associated genes were successively excavated in the TCGA array-Agilent training data set. [file 9915312.f1.zip › 9915312.f1/supplementaty-figure 4 revised.jpg]

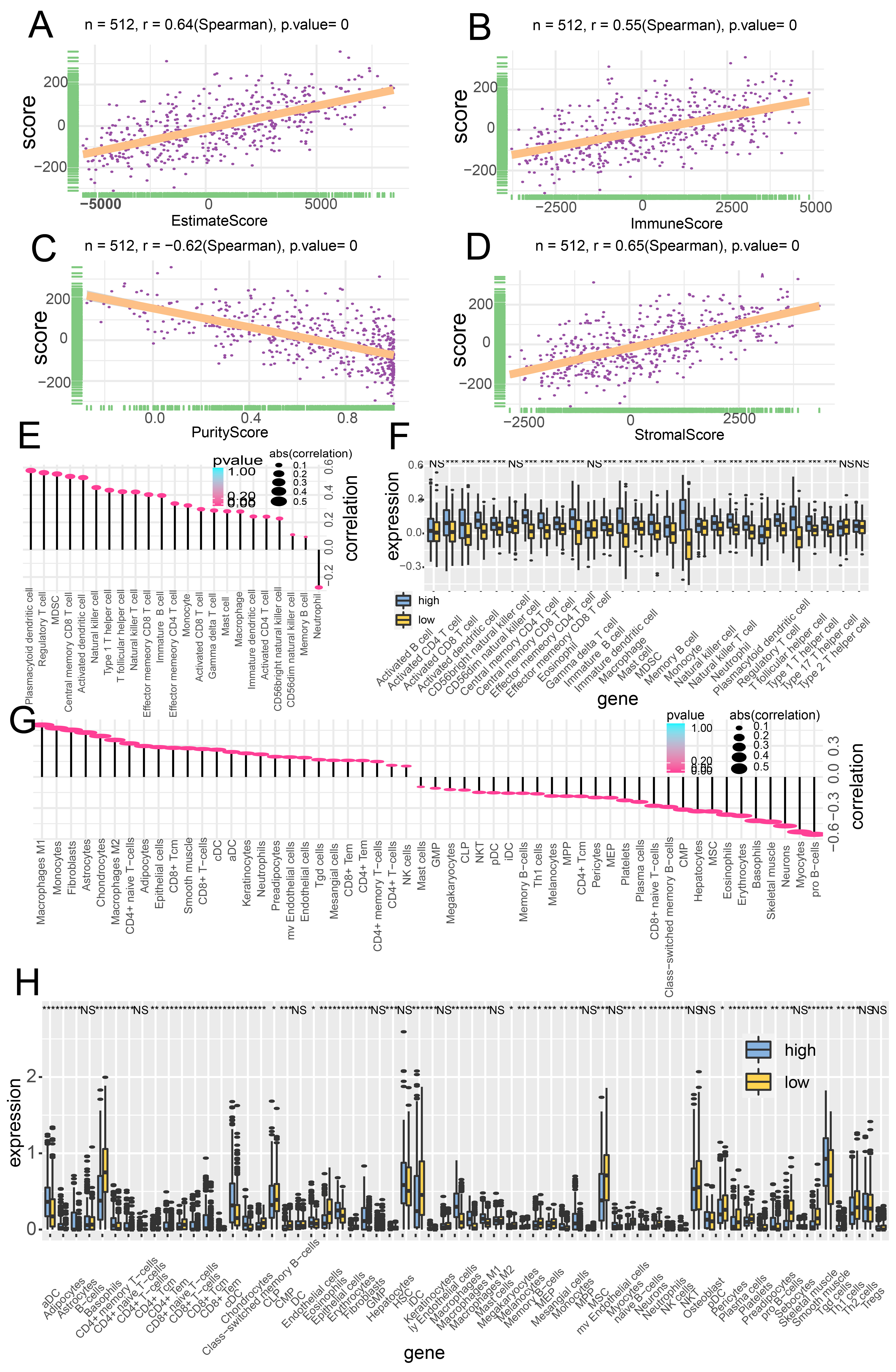

Supplement: Supplementary Materials — Supplementary Figure 1: the workflow of this article. Supplementary Figure 2: univariate Cox regression and LASSO regression were used to establish a risk score model containing 13 ferroptosis-related genes. Supplementary Figure 3: heat map and survival analysis of TCGA seq data set and ROC curve of TCGA array-Agilent data set. Supplementary Figure 4: immune infiltration analysis based on score signature in TCGA seq data set. Supplementary Figure 5: immune infiltration analysis based on score signature in TCGA array-u133a data set. Supplementary Table1: Cancer-progression-associated genes were successively excavated in the TCGA array-Agilent training data set. [file 9915312.f1.zip › 9915312.f1/supplementaty-figure 5 revised.jpg]

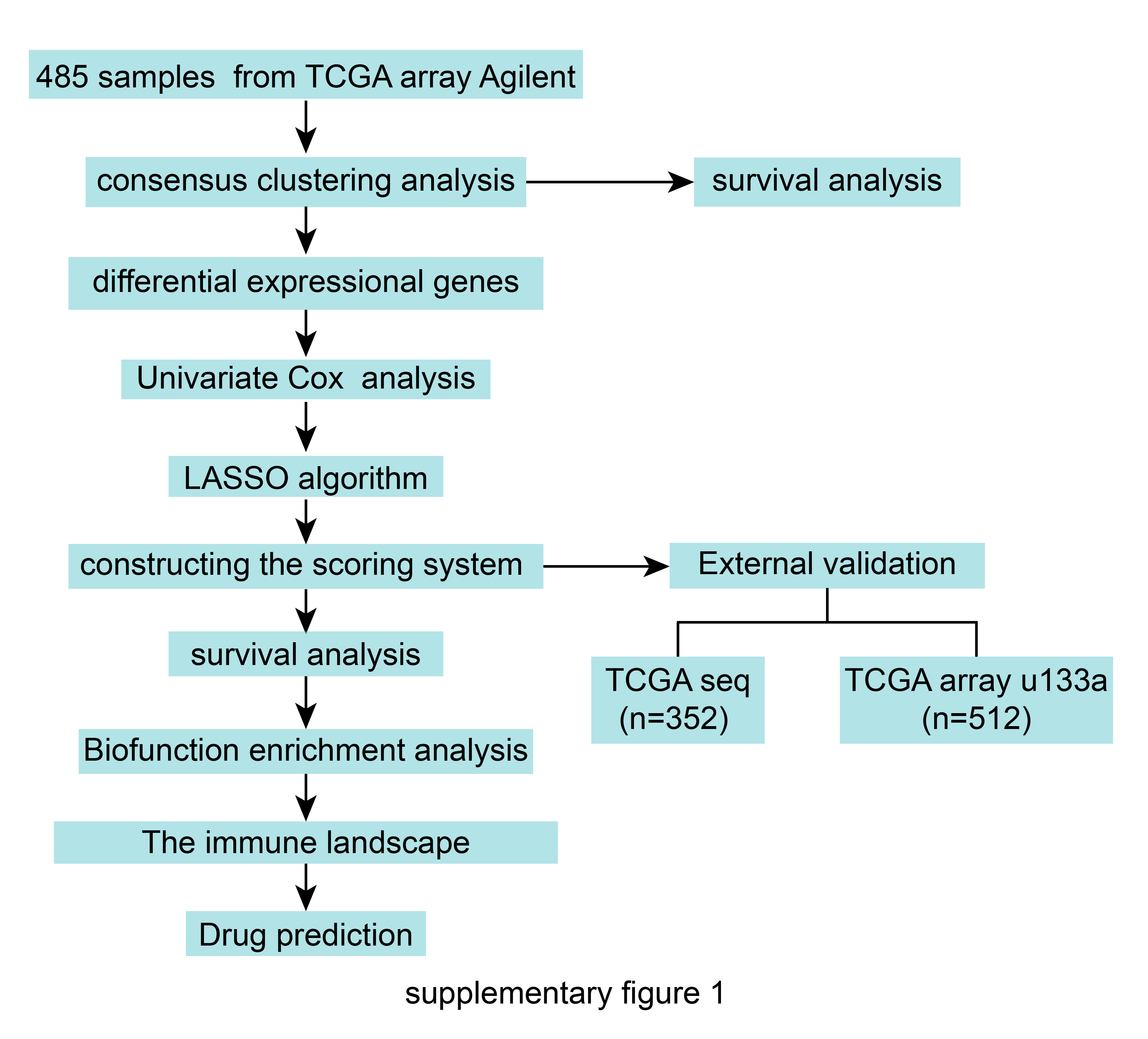

Supplement: Supplementary Materials — Supplementary Figure 1: the workflow of this article. Supplementary Figure 2: univariate Cox regression and LASSO regression were used to establish a risk score model containing 13 ferroptosis-related genes. Supplementary Figure 3: heat map and survival analysis of TCGA seq data set and ROC curve of TCGA array-Agilent data set. Supplementary Figure 4: immune infiltration analysis based on score signature in TCGA seq data set. Supplementary Figure 5: immune infiltration analysis based on score signature in TCGA array-u133a data set. Supplementary Table1: Cancer-progression-associated genes were successively excavated in the TCGA array-Agilent training data set. [file 9915312.f1.zip › 9915312.f1/supplementaty-figure1 revised.jpg]
